# Application of Immune Checkpoint Inhibitors in Cancer

**DOI:** 10.1002/mco2.70176

**Published:** 2025-08-10

**Authors:** Zhijun Chen, Zihan Song, Shichen Den, Wei Zhang, Mengjia Han, Tianhang Lan, Xiaokang Du, Jingyun Ning, XinHui Chen, Haoming Lin, Rui Zhang

**Affiliations:** ^1^ Department of Biliary‐Pancreatic Surgery Sun Yat‐sen Memorial Hospital Sun Yat‐sen University Guangzhou China; ^2^ Department of Liver Surgery Sun Yat‐Sen University Cancer Center Sun Yat‐sen University Guangzhou China; ^3^ Department of Otorhinolaryngology Sun Yat‐Sen University Cancer Center Sun Yat‐sen University Guangzhou China; ^4^ Department of Thoracic Surgery Sun Yat‐Sen University Cancer Center Sun Yat‐sen University Guangzhou China; ^5^ Breast Tumor Center Sun Yat‐Sen University Cancer Center Sun Yat‐sen University Guangzhou China; ^6^ Department of Urology Sun Yat‐Sen University Cancer Center Sun Yat‐sen University Guangzhou China; ^7^ Department of Thoracic Surgery First People's Hospital of Foshan Foshan China

**Keywords:** Adverse events, Advanced malignancies, Clinical application, Immune checkpoints, Immune checkpoint inhibitors

## Abstract

Cancer is a significant challenge to society and public health in the 21st century. According to GLOBOCAN 2020, there were 19.3 million new cancer cases with approximately 10.0 million deaths in 2020 globally. By 2040, 28 million new cases and 16.2 million deaths are estimated. With the escalating challenges of cancer and limitations of conventional therapies like surgery, chemotherapy, and radiotherapy, the development of novel therapies such as immunotherapy and targeted therapy is required. Immunotherapy, especially immune checkpoint inhibitors (ICIs), has become a significant advancement in cancer treatment, combating tumors by activating the immune system. This review offers a thorough overview of ICIs, including their classification, mechanisms of action, and adverse events. It also examines the application of ICIs across various cancer types especially on advanced or unresectable malignancies, such as head and neck squamous cell carcinoma, esophageal cancer, non‐small cell lung cancer, breast cancer, hepatocellular carcinoma, and bladder cancer, highlighting their therapeutic potential and the challenges they face. By providing a comprehensive analysis, this review aims to construct a reference system for clinicians to better understand and utilize ICIs in treating cancer.

## Graphical abstract

Immune checkpoint inhibitors (ICIs) revolutionize oncology by harnessing immunity against advanced cancers. This review systematically explores ICI mechanisms, clinical applications in advanced malignancies (e.g., head and neck squamous cell carcinoma, esophageal cancer, non‐small cell lung cancer, breast cancer, hepatocellular carcinoma, and bladder cancer), and strategies to address immune‐related adverse events (irAEs), providing a critical reference for optimizing immunotherapy in clinical practice.

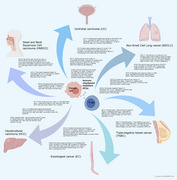

## 1 Introduction

Cancer is a significant challenge to society and public health in the 21st century, accounting for 16.8% of global deaths and 30.3% of premature deaths from noncommunicable diseases [1]. Estimations suggested 28 million new cancer cases and 16.2 million cancer deaths worldwide by 2040 [2]. Traditional treatment methods, including surgery, chemotherapy, and radiotherapy (RT), are the mainstays of cancer management [3]. Surgery is the optimal approach for treating cancer, but it is often not suitable for patients with advanced or unresectable cancer, for whom systemic therapy is the only recourse. However, the therapeutic effects of chemotherapy and RT are limited and can be detrimental to normal cells [3]. In light of the grim landscape of cancer treatment, immunotherapy and targeted therapy have gradually emerged, with immune checkpoint inhibitors (ICIs) becoming one of the effective means for treatment of various cancers.

The development of ICIs spans nearly 40 years. Brunet et al. [4] identified the first inhibitory immune checkpoint—cytotoxic T lymphocyte‐associated antigen 4 (CTLA‐4), marking the start of research on ICIs. Leach et al. [5] discovered the antitumor effect of anti‐CTLA‐4 antibodies in mice, providing the first ever evidence for the application of anti‐CTLA‐4 antibodies in cancer treatment. Subsequently, programmed cell death protein 1 (PD‐1) and B7‐H1 (later confirmed to be programmed cell death protein ligand 1, PD‐L1), along with their antitumor effects, were discovered in later studies [6, 7, 8]. In 2011, the CTLA‐4 inhibitor ipilimumab was approved by the United States Food and Drug Administration (US FDA) for the treatment of unresectable or metastatic melanoma, becoming the world's first approved ICI [9]. PD‐1 inhibitors Nivolumab and PD‐L1 inhibitors atezolizumab were also approved by the US FDA for the treatment of malignant tumors in 2014 and 2016, respectively [10]. [11],. Research on ICIs has progressed rapidly, following the success of PD‐1/PD‐L1 and CTLA‐4 antibody agents, researchers have discovered additional immune checkpoints, such as lymphocyte activation gene 3 (LAG‐3), T cell immunoglobulin mucin 3 (TIM‐3), T cell immunoglobulin and ITIM domain (TIGIT), V‐domain Ig suppressor of T cell activation (VISTA), the nucleotidase family of CD39, CD73, CD38, as well as B7‐H3, B7‐H4 [12]. Preclinical and clinical studies targeting these checkpoints are underway, providing new strategies and hope for cancer treatment. The successful application of ICIs marks a significant advancement in the field of cancer treatment, as they activate the immune system to combat tumors, offering new therapeutic approaches compared with traditional treatments such as chemotherapy and RT.

Although ICIs have significantly improved cancer treatment outcomes, not all patients respond to treatment. Factors such as the immune cell status in the tumor microenvironment (TME), genetic mutations in tumor cells, and the overall health status of patients can all affect the efficacy of ICIs [13, 14, 15, 16]. Tumors can be classified into three immune phenotypes based on the TME: immune‐infiltrated, immune‐excluded, and immune‐desert [17]. The immune‐infiltrated type, also known as “hot” tumors, may respond better to ICIs due to their high level of T cell infiltration, increased PD‐L1 expression, and high tumor mutational burden (TMB) [14]. Genetic alterations in tumor cells, such as microsatellite instability‐high (MSI‐H), mismatch repair deficient (dMMR), and high TMB, favor ICIs therapy and are approved by the US FDA as positive predictive biomarkers for treatment response [15, 16]. Various genetic signals that may serve as predictive biomarkers, such as alterations in DNA damage response genes, MHC‐I genotypes, β‐2‐microglobulin deficiency, and Janus kinase 1/2 (JAK1/2) mutations, are being actively explored [16]. The overall health status of patients, including baseline lymphocyte counts and changes in the gut microbiome, can also affect ICIs’ efficacy and toxicity [18]. Immune‐related adverse events (irAEs) are also a limiting factor for ICI therapy. Therefore, the clinical application of ICIs in cancer treatment is promising but remains challenging.

**FIGURE 1:**
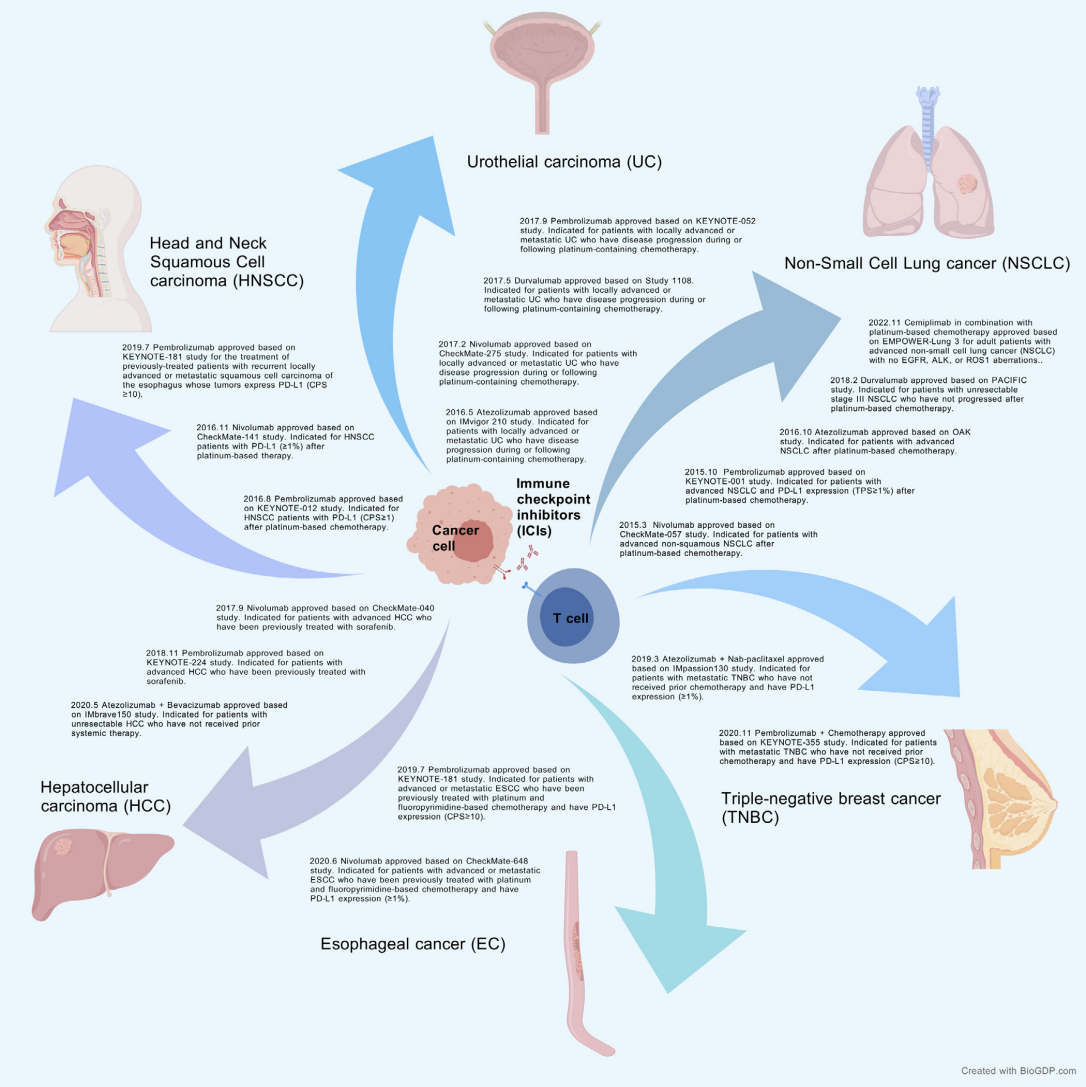
Therapeutic landscape of immune checkpoint inhibitors in diverse cancers. [Created with the Generic Diagramming Platform (GDP, https://BioGDP.com).] This figure summarizes the regulatory approvals of immune checkpoint inhibitors (ICIs) for head and neck squamous cell carcinoma (HNSCC), esophageal cancer (EC), non‐small cell lung cancer (NSCLC), triple‐negative breast cancer (TNBC), hepatocellular carcinoma (HCC), and urothelial carcinoma (UC). The timeline highlights drug names, approval dates, pivotal clinical trials, and indications (including PD‐L1 expression thresholds and prior therapy requirements). This figure is sourced from the official website of the US Food and Drug Administration (US FDA).

This review commences with a classification of ICIs, providing a detailed exposition of the various types of ICIs, their mechanisms of action and related irAEs. We then review the applications of ICIs in different cancers in anatomical order, with special attention given to advanced or unresectable head and neck cancer, esophageal cancer (EC), lung cancer, breast cancer, liver cancer, and bladder cancer. The current approval landscape of ICIs across multiple malignancies is comprehensively summarized in Figure 1. We hope to introduce a comprehensive reference system for clinicians to enhance their understanding of the therapeutic potential and challenges associated with ICIs.

## 2 Immune Checkpoint Inhibitors

Immune checkpoint molecules are expressed on various immune cells and play an important role in regulating immune responses, capable of both activating and suppressing immune reactions [19]. Based on their mechanisms of action, immune checkpoints can generally be divided into costimulatory and coinhibitory receptors [20]. As the name suggests, costimulatory receptors play a crucial role in the activation and proliferation of T cells, and currently discovered costimulatory receptors encompass ICOS, CD226, OX‐40, 4‐1BB, CD28, GITR, and so on [20, 21, 22]. Conversely, coinhibitory receptors, like CTLA‐4, TIGIT, PD‐1, TIM‐3, and LAG‐3, are found to limit T cell activity, preventing excessive immune response [20, 21]. ICIs, monoclonal antibodies targeting the forementioned proteins, interrupting the binding of checkpoints to their ligands, thereby reactivating immune cells to exert antitumor effects [23]. ICIs that are well studied and clinically applied are listed and explained below.

### 2.1 CTLA‐4 Inhibitors

CTLA‐4, similar to CD28 in sequence and genetic position, can recognize and competitively bind on costimulatory ligands B7‐1 (CD80) and B7‐2 (CD86) expressed on antigen‐presenting cells (APCs) [24]. CTLA‐4 inhibits T cell activation through various mechanisms, ranging from competitive binding with CD28 to B7 ligands, inhibiting signal transduction of the T cell receptor (TCR), altering the CD28 positioning in the immunological synapse, to isolating B7 ligands from APCs through transendocytosis, thus inhibiting T cell activation [24].

The commonly seen CTLA‐4 inhibitors are ipilimumab and tremelimumab. Ipilimumab (also known as MDX‐010 and BMS‐734016) was the first ICI approved by the US FDA for the treatment of melanoma. Ipilimumab utilizes IgG1, which shows higher affinity for FcγR and is adept at antibody‐dependent cellular cytotoxicity; it also contributes to the depletion of regulatory T (Treg) cells [24]. Tremelimumab is a human IgG2 CTLA‐4 monoclonal antibody, which has been found to enhance the production of interleukin‐2 (IL‐2) and interferon (INF)‐γ [25]. In 2022, tremelimumab combined with durvalumab was approved for the first time in the treatment of unresectable hepatocellular carcinoma (HCC) patients, while tremelimumab combined with durvalumab and platinum‐based chemotherapy was approved for the treatment of mNSCLC in adult patients without EGFR sensitizing mutations or anaplastic lymphoma kinase (ALK) fusion [25]. Recently, Tang et al. [26] found that HBM4003 (a new anti‐CTLA‐4 antibody) showed good tolerability and antitumor effect in phase I clinical trials, providing a new option for the treatment of advanced melanoma and solid tumors. Nevertheless, the long‐term efficacy and safety of HBM4003 still need to be further verified in future studies.

Ipilimumab's clinical trials unveiled “autoimmune” related adverse events, which were later termed irAEs [24]. The onset and resolution times of irAEs caused by CTLA‐4 inhibitors vary depending on the affected organs, with skin‐related irAEs usually appearing the earliest and resolving quickly, while endocrine‐related irAEs resolve more slowly and may be irreversible in some cases [27]. Skin‐related irAEs include rashes, vitiligo, alopecia areata, and so on, with rashes being the most frequent [28]. Biopsy shows severe dermatitis with dermal edema, sometimes accompanied by perivascular lymphocytic infiltration [29]. In addition, gastrointestinal irAEs caused by CTLA‐4 inhibitors should not be neglected, with reports of fatal intestinal perforation and subsequent sepsis [27]. The most commonly reported endocrine‐related irAE is hypophysitis [27]. Liver enzyme elevation, autoimmune hepatitis, and other liver damage events, as well as conjunctivitis, scleritis, and other ocular irAEs, although less common, also require attention [27, 29, 30]. Tarhini et al. [30] found that rashes and endocrine‐related irAEs in cohorts treated with CTLA‐4 inhibitors are independent prognostic factors determining overall survival (OS) and recurrence‐free survival (RFS), with patients who develop grade 1–2 irAEs having a better OS. Interestingly, the risk of irAEs from CTLA‐4 inhibitors is dose dependent, with higher doses leading to an increased occurrence of irAEs [31]. Moreover, patients receiving CTLA‐4 inhibitors seem to be more susceptible to irAEs than those treated with PD‐1 or PD‐L1 inhibitors [32].

### 2.2 PD‐1/PD‐L1 Inhibitors

PD‐1 (also known as CD279) is expressed in cells of the innate and adaptive immune systems, whereas PD‐L1 and PD‐L2 are expressed in cancer cells and APCs in the TME [33]. In the immune system, binding of PD‐L1 to PD‐1 mainly plays an immunosuppressive regulatory role, thereby weakening the activity of T cells [34]. Tumors evade host immune surveillance by expressing PD‐L1, inducing T cell apoptosis through interaction with PD‐1, thus inhibiting immune response [35]. The combination of PD‐1 and PD‐L1 mainly promotes immune evasion and tumor metastasis through the following mechanisms: (1) The interaction between PD‐1 and PD‐L1 inhibits the activation and proliferation of T cells, leading to T cell dysfunction and apoptosis; (2) This interaction enhances the activity of Treg cells and induces immune tolerance; (3) The interaction encourages the polarization of tumor‐associated macrophages and other immune cells to tumor‐enhancing phenotypes, inducing immune evasion and cancer progression; (4) The signaling of PD‐L1 may prevent apoptosis of tumor cells [36].

The PD‐1 inhibitors currently approved by the US FDA include cemiplimab, dostarlimab, pembrolizumab, nivolumab, retifanlimab, tislelizumab, toripalimab, and so on, while PD‐L1 inhibitors include atezolizumab, avelumab, durvalumab, and so on [37]. IrAEs of PD‐1/PD‐L1 inhibitors throughout treatment are usually insidious and have a wide toxicity spectrum. Relevant studies have found that grade IV‐V irAEs occur earlier than mild irAEs, patients with poor performance status, elevated neutrophil/lymphocyte ratio (NLR), and lung cancer are at higher risk of severe irAEs [38]. It is also discovered that compared with PD‐1 inhibitors, PD‐L1 inhibitors have relatively safe toxicity profiles [37]. Pneumonia, hypothyroidism, arthralgia, and vitiligo are common irAEs in PD‐1 inhibitors [39]. In addition, PD‐1/PD‐L1 inhibitors have been linked to a higher chance of developing periodontitis, which is related to a better survival rate and skin irAEs [40]. Latest research has found that high levels of acylcarnitines and steroid hormone metabolites may be risk factors for irAEs after PD‐1/PD‐L1 inhibitors treatment, these substance levels can also be used to predict OS following treatment [41].

### 2.3 Other Agents

Scientists have identified natural chemical compounds that can influence PD‐1/PD‐L1 pathway. For instance, berberine, a plant‐derived chemical, selectively binds to glutamate 76 of the constitutive photomorphogenesis 9 signalosome 5 and inhibits the PD‐1/PD‐L1 pathway through its deubiquitination activity, leading to degradation of PD‐L1 [42]. Other plant‐derived natural chemicals such as evodiamine, apigenin, cinnamaldehyde, gallic acid, luteolin, myricetin, curcumin, and lycopene can also suppress PD‐L1 expression through various mechanisms [35]. Certain microbial agents, like mithramycin A, when used in combination with PD‐L1 inhibitors, demonstrated to enhance CD8^+^ T cell infiltration around tumors and suppress tumor growth in mouse models [43]. Experiments have revealed that small molecule agents, such as demethylzeylasteral, can significantly downregulate PD‐L1 expression in tumor cells and enhance the cytotoxic effect of T cells on tumor cells, potentially enhancing the efficacy of immunotherapy when used in conjunction with CTLA‐4 antibodies [44]. The utilization of these natural compounds or small molecules, either as monotherapies or combination therapies, presents innovative avenues for the field of ICIs therapy. These discoveries not only enrich the selection of immunotherapeutic agents but also provide new treatment options for patients who are not responsive to traditional ICIs.

### 2.4 Developing ICIs

Studies are underway to uncover the potential of novel ICIs, including those targeting LAG‐3, TIGIT, TIM‐3, VISTA, and B7‐H3, which have exhibited antitumor effects in preclinical and early clinical trial settings.

LAG‐3/CD223 plays an important role in T cell dysfunction and is known to interact with several ligands ranging from galectin‐3 (Gal‐3), liver and lymph node sinusoidal endothelial cell C‐type lectin (LSECtin), fibrinogen‐like protein 1 (FGL1), α‐synuclein preformed fibrils (α‐syn PFF), to TCR–CD3 complex, but further validation is required [45]. LAG‐3 inhibitors have emerged as the latest class of ICIs demonstrating clinical efficacy [45]. Despite suboptimal outcomes with monotherapy, the combination of relatlimab (LAG‐3 inhibitor) and nivolumab (Opdualag) was approved for the management of unresectable or metastatic melanoma in 2022, marking a significant milestone [45]. Kelly et al. [46] demonstrated the first phase Ib clinical trial incorporating PD‐1 and LAG‐3 inhibition with chemoradiotherapy (CRT) for resectable esophageal/gastroesophageal junction cancer, suggesting a potential improvement in 2‐year RFS and OS rates with the addition of relatlimab to nivolumab‐based CRT. Recent researches have shown how the combined use of PD‐1 and LAG‐3 inhibitors may enhance antitumor immunity. Blockade of LAG‐3 and PD‐1 can lead to the coexpression of cytotoxic and exhaustion gene modules in CD8+ T cells to enhance antitumor immunity, though they also synergistically act on driving T cell exhaustion and hindering autocrine IFN‐γ‐dependent antitumor immunity [47, 48].

TIM‐3 was found in 2001 during a research on asthma susceptibility genes in congenic mice [49]. Four TIM‐3 ligands have been identified to date, including galectin‐9 (Gal‐9), high‐mobility group protein B1 (HMGB1), phosphatidylserine (PtdSer), and carcinoembryonic antigen cell adhesion molecule 1 (CEACAM‐1), with Gal‐9 being the most studied [49]. Ma et al. [50] identified the compound ML‐T7 that targets Tim‐3, the combined therapy of ML‐T7 and PD‐1 inhibitors showed superior efficacy to monotherapy in mice, supporting further development of ML‐T7 for antitumor immunotherapy. TIM‐3 stabilization occurs through palmitoylation at cysteine 296 (Cys296) by the enzyme DHHC9, which prevents TIM‐3 polyubiquitination and degradation [51]. It has also been found in liver tumor cells that DHHC9 expression is associated with TIM‐3 expression in CD8+ T cells and NK cells, and high DHHC9 expression correlates with shorter survival in patients with high TIM‐3 expression [51]. Additionally, TIM‐3 inhibitor sabatolimab (MBG453) in combination with PD‐1 inhibitors and iTIM‐3 inhibitor LY3321367 with PD‐L1 inhibitors have shown preliminary antitumor effects in early clinical trials for certain tumors [52, 53]. These results indicate the potential of combination therapy of TIM‐3 inhibitors with PD‐1/PD‐L1 inhibitors, providing new therapeutic approaches for cancer patients.

TIGIT, as a newly discovered immune checkpoint molecule, is present on the surface of lymphocytes. TIGIT is currently believed to have five ligands: CD155, CD112, CD113, Nectin4, and Fab2, playing a significant role in cellular immune suppression [49]. Guan et al. [54] found in mice that aTIGIT inhibitors reshape the TME through FcγR, thereby promoting the transition of antitumor CD8+ T cells from an exhausted effector state to a memory‐like state. Additionally, Piovesan et al. [55] discovered that the combination of Fc‐enabled or Fc‐silent TIGIT inhibitors with PD‐1 inhibitors in mice can enhance tumor control through various mechanisms. Therefore, Fc status is a prominent consideration in the development of TIGIT inhibitors. Currently, several TIGIT inhibitors have been developed in lung cancer and other solid cancers, such as tiragolumab, vibostolimab, and ociperlimab [56, 57, 58]. Early‐phase clinical trials for these novel antibodies have shown promising efficacy and good tolerability. Further study is required to determine their long‐term efficacy, safety, and optimal therapeutic strategies.

VISTA, a member of the B7 family of type I transmembrane immune modulatory glycoproteins, also referred to as PD‐1H or B7‐H5, is the target of the monoclonal antibody KVA12123, which is undergoing phase 1/2 clinical trials for evaluation as a monotherapy or combined with pembrolizumab for advanced solid tumors [59]. Additionally, enoblituzumab, a humanized monoclonal antibody targeting B7‐H3, demonstrated acceptable safety and antitumor effect when combined with pembrolizumab in early clinical trials for head and neck squamous cell carcinoma (HNSCC) and non‐small cell lung cancer (NSCLC) [60]. Gitto et al. [61] found the expression of B7‐H4 in ovarian cancer patients with resistant to platinum and PARPi, and further animal experiments demonstrated that B7‐H4 antibody–drug conjugates (ADCs) can inhibit the growth of B7‐H4‐positive tumors. Research into additional ICIs continues, with these novel agents offering new therapeutic approaches, improving outcomes for patients.

## 3 Therapeutic Landscape of ICIs in Diverse Cancers

### 3.1 Head and Neck Squamous Cell Carcinoma

Head and neck cancer ranks as the sixth most prevalent cancer globally from global cancer statistics 2020, predominantly squamous cell carcinomas [62]. More than 60% of HNSCC patients are diagnosed at an advanced stage, limiting therapeutic options thus worsening prognosis [63]. Despite progress in surgical techniques, medicine development, and CRT, the 5‐year survival rate of HNSCC patients has not seen substantial improvement over recent decades, particularly for HPV‐negative HNSCC [64, 65]. Recently, immunotherapy has emerged as a pivotal aspect in the management of HNSCC, encompassing ICIs, oncolytic virus therapy and chimeric antigen receptor T‐cell (CAR‐T) therapy [66]. Among these options, ICIs are one of the widely utilized immunotherapeutic strategies. Pembrolizumab and nivolumab are currently recommended as standard treatments for recurrent or metastatic (R/M‐) HNSCC, with a number of studies investigating the use of ICIs for locally advanced (LA‐) HNSCC (For comprehensive clinical trial data on HNSCC, please refer to Table 1.) [67].

**TABLE 1:** Clinical trials of ICIs for head and neck squamous cell carcinoma.

Tumor type	Clinical trial name	Registration number	Country	Completion date	Starting date	LOT	Phase	Combination type	Regimens	No. of patients	mPFS (months)	mOS (months)	ORR	Grade 3/4 TRAEs
**Clinical trials with results of ICIs for head and neck squamous cell carcinoma (HNSCC)**														
R/M‐HNSCC	KEYNOTE‐012	NCT01848834	United States	2016/4/26	2013/5/7	Second‐line	Ib	Single‐agent	Pembrolizumab	104	18.0	2.0	13.0%	17.0%
R/M‐HNSCC	CheckMate‐141	NCT02105636	Multiple countries	2021/9/10	2014/5/29	Second‐line	III	Single‐agent	Nivolumab vs. standard therapy (methotrexate, docetaxel, or cetuximab)	361	2.0 vs. 2.3	7.5 vs. 5.1	13.3 vs. 5.8%	13.1%
R/M‐HNSCC	KEYNOTE‐040	NCT02252042	Multiple countries	2022/8/15	2014/11/17	Second‐line	III	Single‐agent	Pembrolizumab vs. standard therapy (methotrexate, docetaxel, or cetuximab)	495	2.1 vs. 2.3	8.4 vs. 6.9	14.6 vs. 10.1%	13.0 vs. 36.0%
LA‐HNSCC	—	NCT02296684	United States	2022/4/5	2015/3/25	NA	II	Single‐agent	Pembrolizumab	36	NA	NA	NA	0.0%
LA‐HNSCC	—	NCT02641093	United States	2021/4/16	2016/1/1	NA	II	Single‐agent	Pembrolizumab	92	NA	NA	NA	62.0%
R/M‐HNSCC	KEYNOTE‐048	NCT02358031	Multiple countries	2023/7/19	2015/3/19	First‐line	III	Single‐agent	Pembrolizumab vs. cetuximab + platinum + 5‐fluorouracil	882	2.3 vs. 5.3	11.5 vs. 10.7	16.9 vs. 36.0%	17.0 vs. 69.3%
								Chemotherapy + ICI (anti‐PD‐1)	Cisplatin/carboplatin + 5‐FU + pembrolizumab vs. cetuximab + platinum + 5‐fluorouracil		4.9 vs. 5.3	13.0 vs. 10.7	37.0 vs. 36.0%	71.7 vs. 69.3%
LA‐HNSCC	—	NCT03342911	United States	2020/10/6	2017/11/13	NA	II	Chemotherapy + ICI (anti‐PD‐1)	Carboplatin + paclitaxel + nivolumab	27	NA	NA	NA	41.0%
LA‐HNSCC	—	NCT04947241	China	2026/1/15	2020/12/15	NA	Ib	Chemotherapy + ICI (anti‐PD‐1)	Gemcitabine + cisplatin + toripalimab	23	NA	NA	45.0%	13.0%
LA‐HNSCC	—	ChiCTR1900025303	China	NA	2019/8/22	NA	II	Chemotherapy + ICI (anti‐PD‐1)	Paclitaxel + docetaxel + camrelizumab	30	NA	NA	96.7%	NA
LA‐HNSCC	—	NCT02919683	United States	2019/7/1	2016/11/1	NA	II	Dual‐ICIs (anti‐PD‐1 + anti‐CTLA‐4)	Nivolumab + ipilimumab vs. nivolumab	29	NA	NA	13.0 vs. 38.0%	NA
R/M‐HNSCC	—	NCT03005782	Multiple countries	2024/4/2	2016/11/7	NA	I	Dual‐ICIs (anti‐PD‐1 + anti‐LAG‐3)	Cemiplimab + fianlimab (ICIs‐naïve population vs. ICIs‐experienced population)	30	NA	NA	7.0 vs. 33.0%	47.0%
LA‐HNSCC	—	NCT03003637	Netherlands	2021/2/12	2017/2/28	NA	Ib/IIa	Dual‐ICIs (anti‐PD‐1 + anti‐CTLA‐4)	Nivolumab + ipilimumab vs. nivolumab	32	NA	NA	NA	NA
HNSCC	—	NCT03247712	United States	Ongoing	2018/1/15	NA	Ib	RT + ICI (anti‐PD‐1)	Radiotherapy + nivolumab	21	NA	NA	NA	19.0%
R/M‐HNSCC	—	NCT02501096	Multiple countries	2023/7/19	2015/3/19	NA	Ib/II	Targeted therapy (TKI) + ICI (anti‐PD‐1)	Lenvatinib + pembrolizumab	22	4.7	NA	46.0%	NA
R/M‐HNSCC	KEYNOTE‐010	NCT04199104	Multiple countries	2025‐03‐31	2020/2/5	First‐line	III	Targeted therapy (TKI) + ICI (anti‐PD‐1)	Lenvatinib + pembrolizumab vs. pembrolizumab	511	6.2 vs. 2.8	15 vs. 17.9	46.1 vs. 25.4%	59.8 vs. 35.2%
R/M‐HNSCC	—	NCT03082534	United States	2024/5/1	2017/3/28	NA	II	Targeted therapy (anti‐VEGF) + ICI (anti‐PD‐1)	Cetuximab + pembrolizumab	33	6.5	18.4	45.0%	42.0%
R/M‐HNSCC	—	NCT03370276	United States	2021/9/4	2017/12/20	NA	II	Targeted therapy (anti‐VEGF) + ICI (anti‐PD‐1)	Cetuximab + nivolumab	88	3.4 vs. 6.2	11.4 vs. 20.2	22.0 vs. 37.0%	NA
LA‐HNSCC	—	NCT04393506	China	2023/11/10	2020/4/23	NA	I	Targeted therapy (anti‐VEGF) + ICI (anti‐PD‐1)	Apatinib + camrelizumab	20	NA	NA	NA	0.0%
**Ongoing clinical trials of ICIs for head and neck squamous cell carcinoma (HNSCC)**														
HNSCC	—	NCT03468218	United States	Ongoing	2018/9/18	NA	II	Targeted therapy (anti‐VEGF) + ICI (anti‐PD‐1)	Cabozantinib + pembrolizumab	41	NA	NA	NA	NA
HNSCC	—	NCT04199104	Multiple countries	Ongoing	2020/2/5	NA	III	Targeted therapy (anti‐TKI) + ICI (anti‐PD‐1)	Lenvatinib + pembrolizumab	511	NA	NA	NA	NA
HNSCC	—	NCT04811027	Multiple countries	Ongoing	2021/8/27	NA	II	Dual‐ICIs (anti‐PD‐1 + anti‐LAG‐3)	Pembrolizumab + eftilagimod alpha	171	NA	NA	NA	NA

The trials in the table are arranged in the order of their appearances in the main text. For trials not explicitly discussed in the text, they are ordered chronologically by trial initiation dates (ascending order).

*Data sources*: ClinicalTrials.gov (clinicaltrials.gov), PubMed, Wanfang Data (https://www.wanfangdata.com.cn/), ESMO (European Society for Medical Oncology), and ASCO (American Society of Clinical Oncology—ASCO).

Abbreviations: anti‐VEGF, antivascular endothelial growth factor; Grade 3/4 TRAEs, Grade 3/4 treatment‐related adverse events; HNSCC, head and neck squamous cell carcinoma; LOT, lines of therapy; mOS, median overall survival; mPFS, median progression‐free survival; NA, not available; No. of patients, number of patients; ORR, objective response rate; R/M‐HNSCC, recurrent/metastatic head and neck squamous cell carcinoma; RT, radiotherapy; TKI, tyrosine kinase inhibitor.

#### 3.1.1 ICIs Monotherapy

KEYNOTE‐012 (NCT01848834) was a multicenter, open‐label, phase Ib clinical study assessing the efficacy, safety, and tolerability of pembrolizumab in patients with R/M‐HNSCC [68]. The study reported an ORR of 18%, with median PFS of 2.0 months and median OS of 13 months. Notably, the occurrence of grade 3/4 irAEs was 9%. With the results of KEYNOTE‐012, US FDA approved pembrolizumab for the treatment of R/M‐HNSCC patients with disease progression following platinum‐based chemotherapy in August 2016, marking a notable advancement in the field of immunotherapy for HNSCC.

Subsequently, CheckMate‐141(NCT02105636) and KEYNOTE‐040 (NCT02252042) further validated the efficacy of immunotherapy in the treatment of R/M‐HNSCC [69, 70]. CheckMate‐141 was a phase III randomized study that investigated the efficacy of Nivolumab in a cohort of 361 patients with platinum‐refractory HNSCC. When compared with the standard‐therapy group, which included methotrexate, docetaxel, or cetuximab, the nivolumab group exhibited superior median OS (7.5 vs. 5.1 months; HR = 0.70, *p* = 0.01) and ORR (13.3 vs. 5.8%), as well as a lower incidence of grade 3/4 irAEs (13.1 vs. 35.1%) [71]. However, no significant difference was found in median PFS between the two groups (2 vs. 2.3 months; HR = 0.89, *p* = 0.32) [71]. KEYNOTE‐040 (NCT02252042) was a phase III, randomized, open‐label trial that assessed pembrolizumab's efficacy versus methotrexate, docetaxel or cetuximab in 495 patients with R/M‐HNSCC. The pembrolizumab group showed a significant improvement in OS compared with the standard treatment group (8.4 vs. 6.9 months; HR = 0.80; *p* = 0.016), as well as a higher ORR (14.6 vs. 10.1%) and a lower incidence of grade 3/4 irAEs (13 vs. 36%) [70]. However, no significant difference was found in PFS [70]. Observed survival benefit demonstrated that nivolumab and pembrolizumab are as effective as second‐line therapeutic options, leading to their addition in the NCCN guidelines.

The NCT02296684 study exhibited that pembrolizumab was well tolerated in patients with HPV‐unrelated LA‐HNSCC, with a pathological response observed in 44% of participants [72]. In patients exhibiting high‐risk pathological characteristics, the 1‐year recurrence rate was lower compared with historical data. The NCT02641093 trial was conducted to assess the efficacy of pembrolizumab as a neoadjuvant and adjuvant therapy, combined with standard chemotherapy and RT for resectable LA‐HNSCC [73]. This study revealed that patients with intermediate‐risk to high‐risk pathological features (positive margins, extracapsular spread) exhibited a 1‐year disease‐free survival (DFS) rate of 96% (95% CI, 90–100%) and 66% (95% CI, 55–84%), respectively [73]. Compared with the result of RTOG 9501, the 1‐year DFS rate among patients classified as intermediate‐risk has increased by 27% [73]. Patients exhibiting a pathological response demonstrated a significantly superior 1‐year DFS rate compared with those without (93 versus 72%, *p* = 0.004) [73]. The aforementioned clinical trials indicate that monotherapy utilizing ICIs is both safe and exhibits a promising therapeutic efficacy in LA‐HNSCC patients. Currently, a global, phase III clinical randomized controlled trial KEYNOTE‐689 is underway, which is expected to further elucidate the value of neoadjuvant pembrolizumab in the immunotherapy of LA‐HNSCC patients [74].

#### 3.1.2 Combination with Chemotherapy

KEYNOTE‐048, a global, multicenter, randomized phase III study designed to assess the efficacy and safety of pembrolizumab with or without chemotherapy (cisplatin or carboplatin plus 5‐FU) versus cetuximab with chemotherapy (EXTREME regimen) in 882 patients with previously untreated R/M‐HNSCC [75, 76]. The trial showed an improved median OS and 5‐year OS rates in pembrolizumab monotherapy compared with the EXTREME regimen, especially in the PD‐L1 combined positive score (CPS) ≥ 1 population with improved median OS from 10.4 to 12.3 months, with an increased 5‐year OS rate from 5.5 to 15.4% [75, 76]. Additionally, the combined application of pembrolizumab and chemotherapy significantly improved median OS and 5‐year OS rates in both CPS ≥ 1 and CPS ≥ 20 population [75, 76]. Furthermore, across the overall population, median OS was improved from 10.7 to 13.0 months (HR = 0.72), and the 5‐year OS rate from 5.2 to 16.0% [75, 76]. The incidence of grade 3 or higher irAEs associated with pembrolizumab monotherapy was notably lower compared with the EXTREME regimen (17.0 vs. 69.3%) [75, 76]. Conversely, the rate of irAEs with pembrolizumab combined with chemotherapy was similar to that of the EXTREME regimen (71.7 vs. 69.3%) [75, 76]. These findings support the combined use of pembrolizumab with platinum and 5‐FU as first‐line treatment for R/M‐HNSCC in the overall population and pembrolizumab monotherapy as a first‐line option in patients with PD‐L1 CPS ≥ 1.

The effectiveness and safety of combined immunochemotherapy have been confirmed in R/M‐HSNCC. However, further clinical research is required to substantiate their impact on locally advanced disease. The NCT03342911 trial evaluated the effectiveness of Nivolumab combined with carboplatin and paclitaxel for the treatment of resectable stage III‐IV HNSCC. The trial reported that 42% of patients reached pathologic complete response (pCR), and 69% of patients exhibited either a major pathologic response (MPR) or pCR. Furthermore, 37% of patients experienced grade 3 irAEs [77]. The NCT04947241 trial evaluated the safety and effectiveness of the PD‐1 inhibitor toripalimab in combination with gemcitabine and cisplatin as neoadjuvant treatment for resectable LA‐HNSCC [78]. A total of 23 patients were enrolled and all underwent successful surgical resection, achieving a 100% R0 resection rate [78]. The pCR rate was 16.7%, and the MPR rate was 27.8% [78]. These findings suggest that triweekly neoadjuvant toripalimab‐GP therapy is feasible in patients with LA‐HNSCC with favorable pCR and MPR rates. Another phase II clinical trial (ChiCTR1900025303) demonstrated that neoadjuvant chemotherapy in combination with camrelizumab for LA‐HNSCC achieved a high ORR, MPR, and pCR rate, with acceptable safety profiles [79]. These aforementioned studies indicate promising pathological response rates and safety profiles in neoadjuvant chemoimmunotherapy.

#### 3.1.3 Dual ICIs

The NCT02919683 trial was a phase II randomized clinical study designed to compare the efficacy and safety of neoadjuvant Nivolumab monotherapy (N) versus combination therapy with Nivolumab and Ipilimumab (N+I) in patients with untreated oral squamous cell carcinoma [80]. This trial reported significantly higher MPR rate in patients treated with N+I compared with those receiving N (20 vs. 8%) [80]. Subsequently, the IMCISION trial demonstrated a more favorable MPR rate in N+I than N in HNSCC (35 vs. 17%). Both trials exhibited promising safety profiles, with no compromise to surgical timelines [81]. Notably, in the IMCISION trial, patients who achieved MPR did not experience recurrence within the 2‐year follow‐up. These results indicate that the combination of ICIs is a promising therapeutic approach.

#### 3.1.4 Combined with RT

The phase Ib clinical trial NCT03247712 evaluated the efficacy of immunotherapy combined with stereotactic body RT (SBRT) in 21 patients diagnosed with LA‐HNSCC [82]. The trial showed that all patients successfully completed the treatment without experiencing any treatment‐related surgical delays. Clinical pathological downstaging was achieved in 90% of the patients, and the occurrence of grade 3/4 irAEs was 19.0% (four out of 21) [82]. Among HPV‐positive and HPV‐negative HNSCC patients who received neoadjuvant Nivolumab combined with SBRT, the MPR rates were 100 and 60%, respectively, and the pCR rates were 90 and 20%, respectively [82]. These findings indicate that the combination of neoadjuvant immune‐RT is safe and demonstrates greater benefits in HPV‐positive patients. In a separate retrospective study evaluating the efficacy and safety of the combination of neoadjuvant nivolumab with SBRT for locally advanced oral squamous cell carcinoma (LA‐OCSCC), none of the 30 eligible patients experienced severe irAEs [83]. A total of 27 patients (90.0%) underwent R0 resection, and five patients (16.7%) experienced surgery‐related complications [83]. Clinical downstaging was achieved in 83.3% of patients, with MPR and pCR rates of 60.0 and 33.3%, respectively [83]. A 24‐month DFS rate of 70.4% and a 24‐month OS rate of 76.4% were observed. These findings indicate that neoadjuvant nivolumab combined with SBRT is both safe and effective, potentially offering a viable neoadjuvant treatment option for patients with LA‐HNSCC.

#### 3.1.5 Combined with Targeted Therapy

Two phase II clinical studies evaluating the combined use of PD‐1 inhibitors with EGFR inhibitors have shown that the combination of pembrolizumab or nivolumab with cetuximab exhibits promising efficacy and safety in R/M‐HNSCC, demonstrating its potential as a first‐line treatment option [84, 85]. A phase II single‐arm clinical study investigating the combined use of pembrolizumab with VEGFR inhibitor cabozantinib in 36 patients with R/M‐HNSCC demonstrated a median PFS of 14.6 months and median OS of 22.3 months [86]. A phase Ib/II single‐arm clinical study evaluating the combination of pembrolizumab with multikinase inhibitor lenvatinib (NCT02501096) exhibited significant antitumor activity in patients with R/M‐HNSCC. An interim analysis of a large phase III clinical trial (NCT04199104) comparing pembrolizumab with or without lenvatinib as first‐line treatment for R/M‐HNSCC patients demonstrated significant improvement in ORR (46.1 vs. 25.4%) and PFS (6.2 vs. 2.8 months) with combined use of lenvatinib and pembrolizumab, but not OS (15 vs. 17.9 months; HR = 1.15, *p* = 0.882) when compared with pembrolizumab plus placebo [87]. These findings underscore the need for further studies to identify optimal treatment strategies for R/M‐HNSCC patients.

A pilot study (NCT04393506) was conducted to explore the combined use of the PD‐1 inhibitor camrelizumab and VEGFR2 inhibitor Apatinib in LA‐HNSCC [88]. The treatment regimen demonstrated safety and good tolerability, with a MPR rate of 40% with no grade 3/4 irAEs [88]. Notably, all patients with a CPS ≥ 10 achieved MPR. Further analysis showed a local recurrence rate of 10.5% and a survival rate of 95% at 18 months [88]. These discoveries highlight the potential of ICIs combined with targeted therapy and necessitating further phase II and III clinical trials.

KEYNOTE‐012, CheckMate‐141, and KEYNOTE‐040 are pioneering clinical trials that have profoundly influenced the advancement of immunotherapy in HNSCC. With the successful application of PD‐1 inhibitors in R/M‐HNSCC, immunotherapy is expected to be utilized in locally advanced patients, thereby benefiting a larger population. However, monotherapy with ICIs often shows limited efficacy in HNSCC, especially in R/M‐HNSCC. The observed ORR and PFS are relatively unsignificant, suggesting limited therapeutic benefits of ICIs monotherapy for these patients. Therefore, exploration of combined immunotherapies is needed to achieve better outcomes.

#### 3.1.6 New Immune Checkpoints for HNSCC

Immunotherapy has revolutionized the treatment landscape for head and neck cancers, with ICIs emerging as pivotal agents for HNSCC. CTLA‐4 and PD‐1 ranked as the most extensively studied and clinically validated targets. Current research is actively exploring next‐generation immune checkpoints, including TIGIT, LAG‐3, and TIM‐3, as potential targets to be included in ICIs therapy.

A phase I clinical study investigating the combination of fianlimab (an anti‐LAG‐3 antibody) and cemiplimab (an anti‐PD‐1 antibody) in advanced HNSCC patients revealed a satisfactory level of safety, tolerability, and efficacy [89]. Furthermore, a randomized phase IIb trial is currently in progress to study the effectiveness of Eftilagimod alpha (a soluble LAG‐3 protein) in combination with pembrolizumab as a second‐line treatment for patients with R/M HNSCC [90]. While several clinical studies have been conducted in various cancers, the development of relevant clinical studies in HNSCC is still pending [56, 91]. The combination of TIM‐3 inhibitors with other ICIs has demonstrated potential for synergistic effects thus enhancing antitumor immunity [92], with promising results in preclinical studies and is currently under evaluation in clinical trials [93].

The successful application of monoclonal ICIs in R/M‐HNSCC paves the way for the advancement of immunotherapy to patients with locally advanced disease, potentially extending benefits to a broader patient population. Moving forward, efforts should be directed toward advocating for neoadjuvant immunotherapy in locally advanced HNSCC, carrying out high‐quality researches, refining combination immunotherapy strategies, and achieving precision therapy.

### 3.2 Esophageal Cancer

Global cancer incidence and mortality data from GLOBOCAN 2022 indicate that EC accounts for over 510,716 new diagnoses and more than 445,129 mortalities globally, placing it seventh in global mortality rankings [1]. Because of the absence of symptoms in early‐stage EC, the majority of patients are already in advanced stages when seeking medical attention, resulting in a generally poor prognosis. The 5‐year survival rate for EC stands at 10–30%, plummeting to 5% when distant metastasis is present at diagnosis [94]. We systematically summarize completed and ongoing clinical trials evaluating the application of ICIs in EC, as detailed in Table 2.

**TABLE 2:** Clinical trials of ICIs for esophageal cancer.

Tumor type	Clinical trial name	Registration number	Country	Completion date	Starting date	LOT	Phase	Combination type	Regimens	No. of patients	mPFS (months)	mOS (months)	ORR	Grade 3/4 TRAEs
**Clinical trials with results of ICIs for esophageal cancer (EC)**														
Advanced EC	KEY‐NOTE‐028	NCT02054806	Multiple countries	2021/4/30	2014/2/17	First‐line	I	Single‐agent	Pembrolizumab	477	1.8	7.0	30.0%	17.0%
Advanced EC	CheckMate 577	NCT02743494	Multiple countries	2020/5/12	2016/7/14	Second‐line	III	Single‐agent	Nivolumab	794	22.4	29.4	60.0%	13.0%
Advanced EC	CheckMate649	NCT02872116	Multiple countries	2024/6/6	2016/10/12	First‐line	III	Chemotherapy + ICIs (anti‐PD‐1)	Chemotherapy + nivolumab	2031	8.3	14.4	64.0%	59.0%
Advanced EC	KEYNOTE‐590	NCT03189719	Multiple countries	2023/7/10	2017/7/25	First‐line	III	Chemotherapy + ICIs (anti‐PD‐1)	Cisplatin + 5‐fluorouracil + pembrolizumab	749	6.3	12.3	45.0%	72.0%
Advanced EC	JUPITER‐06	NCT03829969	China	2023/9/30	2019/1/31	First‐line	III	Chemotherapy + ICIs (anti‐PD‐1)	Paclitaxel + cisplatin + toripalimab	514	5.7	17.0	69.3%	73.2%
Advanced EC	ORIENT‐15	NCT03748134	Multiple countries	2023/7/29	2018/12/24	First‐line	III	Chemotherapy + ICIs (anti‐PD‐1)	Chemotherapy + sintilimab	746	7.2	16.7	66.1%	59.9%
Advanced EC	RATIONALE‐302	NCT03430843	Multiple countries	2022/12/28	2018/1/26	Second‐line	III	Chemotherapy + ICIs (anti‐PD‐1)	Chemotherapy + tislelizumab	512	1.6	8.6	20.3%	73.3%
Advanced EC	RATIONALE‐306	NCT03783442	Multiple countries	2024/8/22	2018/12/11	First‐line	III	Chemotherapy + ICIs (anti‐PD‐1)	Chemotherapy + tislelizumab	649	7.3	17.2	63.5%	66.7%
Advanced EC	ESCORT‐1st	NCT03691090	China	2020/10/30	2018/12/3	First‐line	III	Chemotherapy + ICIs (anti‐PD‐1)	Paclitaxel + cisplatin + SHR‐1210	596	6.9	15.3	72.1%	63.4%
Advanced EC	CheckMate648	NCT03143153	Multiple countries	2021/1/18	2017/6/29	First‐line	III	Dual‐ICIs (anti‐PD‐1 + anti‐CTLA‐4)	Nivolumab + ipilimumab	970	2.9	12.8	35.0%	32.0%
**Ongoing clinical trials of ICIs for Esophageal cancer (EC)**														
Advanced EC	KEYNOTE‐975	NCT04210115	Multiple countries	Ongoing	2020/2/28	Potential First‐line	III	dCRT + ICIs (anti‐PD‐1)	Definitive chemoradiotherapy + pembrolizumab	703	NA	NA	NA	NA
Advanced EC	SKYSCRAPER‐07	NCT04543617	Multiple countries	Ongoing	2020/9/28	Potential Second‐line	III	Dual‐ICIs (anti‐PD‐L1 + anti‐TIGIT)	Atezolizumab + tiragolumab	760	NA	NA	NA	NA
Advanced EC	SKYSCRAPER‐08	NCT04540211	Multiple countries	Ongoing	2020/10/30	Potential First‐line	III	Dual‐ICIs (anti‐PD‐L1 + anti‐TIGIT) + chemotherapy	Atezolizumab + tiragolumab + paclitaxel + cisplatin	461	NA	NA	NA	NA

The trials in the table are arranged in the order of their appearances in the main text. For trials not explicitly discussed in the text, they are ordered chronologically by trial initiation dates (ascending order).

*Data sources*: ClinicalTrials.gov (clinicaltrials.gov), PubMed, Wanfang Data (https://www.wanfangdata.com.cn/), ESMO (European Society for Medical Oncology), and ASCO (American Society of Clinical Oncology—ASCO).

Abbreviations: dCRT, definitive chemoradiotherapy; EC, esophageal cancer.

#### 3.2.1 ICIs Monotherapy

The field of EC immunotherapy is advancing. In February 2014, KEYNOTE‐028 marked the beginning of ICIs monotherapy for EC, it is a multicenter, nonrandomized, open‐label study administering pembrolizumab at 10 mg/kg every 3 weeks to patients with PD‐L1‐positive esophageal squamous cell carcinoma (ESCC) or adenocarcinoma [95]. Tumor shrinkage occurred in over half of the patients, with 28% (five out of 18) achieving partial remission (PR) [95]. The median response duration was 15 months, the median PFS was 1.8 months, and the median OS was 7.0 months [95]. Less than 40% of participants reported grade 2 irAEs, primarily skin rash and anorexia, with no grade 4 irAEs observed [95]. The result demonstrated the efficacy of pembrolizumab with a manageable incidence of irAEs, resulted in its US FDA approval as the first PD‐1 inhibitor for EC treatment. In July 2016, CheckMate 577 with 794 participants, investigated Nivolumab's effects on patients without pCR after surgery, showing a median DFS of 22.4 months in the treatment group, compared with 10.4 months in the placebo group (HR = 0.67) [96]. However, grade 3–4 irAEs occurred in 14% of patients, with 8% having severe grade 4 irAEs. Most irAEs were manageable, with skin rash, anorexia, and bone marrow suppression being the most common [96].

#### 3.2.2 Combination with Chemotherapy

Studies such as CheckMate649 and KEYNOTE‐590 have demonstrated significantly improved OS with the combined use of pembrolizumab or nivolumab with platinum‐based chemotherapy in patients with locally advanced or metastatic EC, irrespective of PD‐L1 status [97, 98]. The JUPITER‐06 study, focusing on advanced ESCC, found that combining toripalimab to chemotherapy increased the ORR by 17.2%, with a 1‐year PFS of 42% (HR = 0.58) and a median OS of 17.0 months compared with 11.0 months with chemotherapy (HR = 0.58) [99]. While this study was limited to squamous cell carcinoma and did not account for racial differences, the benefits of toripalimab in combination with chemotherapy were evident [100]. The ORIENT‐15 study initiated in 2018, included Asian patients with ESCC and found that Sintilimab combined with chemotherapy improved the overall ORR to 66.1%, a 20.6% improvement over the 45.5% with chemotherapy alone [101]. The RATIONALE‐302 and RATIONALE‐306 studies validated the effectiveness and safety of tislelizumab in combination with chemotherapy, leading to its NMPA approval of first and second‐line treatment strategy for ESCC [102, 103]. The ESCORT‐1st study, a phase III, prospective, multicenter, randomized controlled, double‐blind trial, included advanced/metastatic ESCC patients demonstrated a higher 3‐year OS rate in the camrelizumab plus chemotherapy group compared with the chemotherapy group, regardless of PD‐L1 status, extending survival beyond 3 years in 25% of patients [104].

#### 3.2.3 Combined with Other Therapies

Concurrent CRT is the standard treatment for patients with unresectable locally advanced ESCC. The combined use of PD‐L1 and CTLA‐4 dual‐ICIs therapy with concurrent CRT has shown promising results, significantly improving OS and PFS in locally advanced ESCC patients [105, 106]. The ongoing KEYNOTE‐975 is investigating the efficacy of pembrolizumab combined with concurrent CRT in 703 patients, examining the effects of immunotherapy in combination with radiation doses of 50 or 60 Gy [107]. It is prominent to consider the impact of concurrent immunotherapy on the incidence of irAEs, given the complex adverse events associated with definitive CRT in EC patients. The SKYSCRAPER‐07 and SKYSCRAPER‐08 trials are also assessing the safety and efficacy of dual immunotherapy of atezolizumab and tislelizumab in advanced EC [108, 109].

In conclusion, ICIs have contributed significantly in treating EC. PD‐L1 status can be utilized to determine potential treatment benefits, though even PD‐L1 negative patients may benefit from immunotherapy. Combining immunotherapy with chemotherapy has been shown to increase pathologic response and survival rates, marking a new era in EC treatment. Immunotherapy's potential in neoadjuvant therapy in resectable ESCC, warrants further exploration [110, 111].

### 3.3 Non‐Small Cell Lung Cancer

Lung cancer, the malignancy with the highest morbidity and mortality rate globally, among which, NSCLC comprises at least 80% of lung cancer patients [1]. In patients with driver gene‐positive mutations, such as EGFR mutations, ALK rearrangements and ROS1 fusions, ICIs have demonstrated limited efficacy in these populations [112]. Conversely, for driver gene‐negative patients, ICIs targeting PD‐1 and PD‐L1 are becoming a significant treatment approach for advanced NSCLC [113, 114]. We have summarized information on completed and ongoing clinical trials about the application of ICIs in NSCLC (see Table 3 for details).

**TABLE 3:** Clinical trials of ICIs for non‐small cell lung cancer.

Tumor type	Clinical trial name	Registration number	Country	Completion date	Starting date	LOT	Phase	Combination type	Regimens	No. of patients	mPFS (months)	mOS (months)	ORR	Grade 3/4 TRAEs
**Clinical trials with results of ICIs for non‐small cell lung cancer (NSCLC)**														
Advanced NSCLC	KEYNOYE‐024	NCT02142738	Multiple countries	2021/5/27	2014/8/25	First‐line	III	Single‐agent	Pembrolizumab	305	10.3	30.0	82.0%	18.0%
Advanced NSCLC	KEYNOYE‐042	NCT02220894	Multiple countries	2022/9/12	2014/10/30	First‐line	III	Single‐agent	Pembrolizumab	1274	7.1	20.0	41.7%	19.5%
Advanced NSCLC	IMpower110	NCT02409342	Multiple countries	2022/3/8	2015/7/20	First‐line	III	Single‐agent	Atezolizumab	572	8.1	20.2	38.3%	30.1%
Advanced NSCLC	EMPOWER‐Lung 1	NCT03088540	Multiple countries	2025/6/30	2017/5/29	First‐line	III	Single‐agent	Cemiplimab	712	8.1	26.1	42.0%	18.0%
Advanced NSCLC	KEYNOTE 010	NCT01905657	Multiple countries	2020/9/30	2013/8/9	Second‐line	II/III	Single‐agent	Pembrolizumab	1034	5.2	17.3	33.1%	16.0%
Advanced NSCLC	OAK	NCT02008227	Multiple countries	2019/1/9	2014/3/11	Second‐line	III	Single‐agent	Atezolizumab	1225	9.6	13.8	14.0%	17.3%
Advanced NSCLC	IMpower130	NCT02367781	Multiple countries	2021/1/18	2015/4/16	First‐line	III	Chemotherapy + ICIs (anti‐PD‐L1)	Atezolizumab + carboplatin‐paclitaxel	723	7.0	18.6	49.2%	81.0%
Advanced NSCLC	KEYNOTE‐189	NCT02578680	Multiple countries	2020/6/22	2016/1/15	First‐line	III	Chemotherapy + ICIs (anti‐PD‐1)	Pemetrexed + platinum + pembrolizumab	616	8.8	22.0	47.6%	67.2%
Advanced NSCLC	KEYNOTE‐407	NCT02775435	Multiple countries	2023/9/14	2016/6/9	First‐line	III	Chemotherapy + ICIs (anti‐PD‐1)	Carboplatin‐paclitaxel + pembrolizumab	559	8.0	17.1	62.6%	69.8%
Advanced NSCLC	CameL	NCT03134872	China	2019/8/29	2017/5/12	First‐line	III	Chemotherapy + ICIs (anti‐PD‐1)	Pemetrexed + carboplatin + SHR‐1210	419	11.3	27.9	60.5%	<1.0%
Advanced NSCLC	RATIONALE 304	NCT03663205	China	2023/4/26	2018/7/23	First‐line	III	Chemotherapy + ICIs (anti‐PD‐1)	Pemetrexed + carboplatin + tislelizumab	334	9.7	21.6	57.0%	31.0%
Advanced NSCLC	ORINET‐11	NCT03607539	China	2023/2/13	2018/8/23	First‐line	III	Chemotherapy +ICIs (anti‐PD‐1)	Pemetrexed + carboplatin + sintilimab	397	9.2	24.2	51.9%	6.1%
Advanced NSCLC	GEMSTONE‐302	NCT03789604	China	2023/5/15	2018/12/13	First‐line	III	Chemotherapy + ICIs (anti‐PD‐1)	Platinum‐containing chemotherapy + CS1001	479	9.0	25.4	64.3%	61.9%
Advanced NSCLC	AK105‐302	NCT03866993	China	2022/1/14	2018/12/20	First‐line	III	Chemotherapy + ICIs (anti‐PD‐1)	Carboplatin + paclitaxel + AK105	350	8.1	NA	71.0%	4.0%
Advanced NSCLC	CHOICE‐01	NCT03856411	China	2023/1/9	2019/3/18	First‐line	III	Chemotherapy + ICIs (anti‐PD‐L1)	Platinum‐containing chemotherapy + toripalimab	465	8.4	23.8	65.7%	78.9%
Advanced NSCLC	ASTRUM‐004	NCT04033354	China	2023/12/30	2019/8/14	First‐line	III	Chemotherapy + ICIs (anti‐PD‐1)	Carboplatin + paclitaxel + HLX10	537	8.3	22.7	62.2%	35.2%
Metastatic nonsquamous NSCLC	IMpower150	NCT02366143	Multiple countries	2020/12/7	2015/3/31	First‐line	III	Chemotherapy + targeted therapy (anti‐VEGF) + ICIs (anti‐PD‐L1)	Carboplatin + paclitaxel + bevacizumab + atezolizumab	1202	8.3	19.2	63.5%	56.7%
Advanced NSCLC	CheckMate‐227	NCT02477826	Multiple countries	2024/10/25	2015/8/5	First‐line	III	Dual‐ICIs (anti‐PD‐1 + anti‐CTLA‐4)	Nivolumab+ipilimumab	2876	5.8	21.0	35.9%	33.0%
Advanced NSCLC	POSEIDON	NCT03164616	Multiple countries	2021/3/12	2017/6/1	First‐line	III	Dual‐ICIs (anti‐PD‐L1 + anti‐CTLA‐4)	Durvalumab + tremelimumab	1013	6.2	14.0	38.8%	53.3%
**Ongoing clinical trials of ICIs for non‐small cell lung cancer (NSCLC)**														
Advanced NSCLC	EVOKE‐02	NCT05186974	Multiple countries	Ongoing	2022/5/30	Potential First‐line	II	Chemotherapy + ADC + ICIs (anti‐PD‐1)	Carboplatin + sacituzumab govitecan (SG) + pembrolizumab	193	NA	NA	NA	NA
Advanced NSCLC	GALLANT‐1	NCT05240131	Multiple countries	Ongoing	2022/3/15	Potential First‐line	I/II	ICIs (anti‐PD‐L1 + anti‐TIGIT)	Atezolizumab+GB1211	88	NA	NA	NA	NA
Advanced NSCLC	TROPION‐LUNG01	NCT04656652	Multiple countries	Ongoing	2020/12/21	Potential Second‐line	III	Chemotherapy + ADC + ICIs (anti‐PD‐L1/anti‐PD‐1)	Docetaxel + datopotamab deruxtecan + ICIs	590	NA	NA	NA	NA

The trials in the table are arranged in the order of their appearances in the main text. For trials not explicitly discussed in the text, they are ordered chronologically by trial initiation dates (ascending order).

*Data sources*: ClinicalTrials.gov (clinicaltrials.gov), PubMed, Wanfang Data (https://www.wanfangdata.com.cn/), ESMO (European Society for Medical Oncology), and ASCO (American Society of Clinical Oncology—ASCO).

Abbreviations: ADC, antibody–drug conjugate; NSCLC, non‐small cell lung cancer.

#### 3.3.1 ICIs Monotherapy

KEYNOTE‐024 and KEYNOTE‐042, initiated in August 2014 and 2015, respectively, marked the beginning of ICIs monotherapy for advanced NSCLC. These trials indicated that pembrolizumab significantly improved ORR and extended PFS in patients with driver gene‐negative advanced NSCLC, irrespective of PD‐L1 tumor proportion score (TPS) being ≥50% or ≥1%, compared with chemotherapy alone [115]. IMpower110, a global, multicenter, randomized phase III study in July 2015, included 572 untreated patients with advanced NSCLC, the majority of whom had driver gene‐negative tumors. The trial demonstrated significantly improved PFS (8.2 months) and OS (20.2 months) with atezolizumab in population with high PD‐L1 expression, compared with chemotherapy alone [116]. Meanwhile, EMPOW‐ER‐Lung 1, a multicenter, open‐label, randomized, phase III study in May 2017, broadened the choice of ICIs monotherapy in advanced NSCLC. The study enrolled 712 patients and exhibited an ORR of 42% and a median OS of 26.1 months with HR = 0.57 [117]. For second‐line treatment of advanced NSCLC, data from the clinical studies of KEYNOTE 010 and OAK showed that pembrolizumab and atezolizumab were able to improve the median OS of patients (KEYNOTE 010: mOS 17.3 vs. 8.2 months; OAK: mOS 13.8 vs. 9.6 months; HR = 0.74) [118, 119]. While in driver gene‐positive patients with disease progression who had undergone platinum‐based chemotherapy, ICIs monotherapy has better outcomes when compared with the subsequent‐line chemotherapy regimen docetaxel [120, 121]. However, from the above studies, ICIs monotherapy is recommended for patients with PD‐L1 TPS ≥50%, for those with low or no expression, the benefit of immunotherapy is low. Therefore, further studies of immunotherapy combination regimens are required.

#### 3.3.2 Combination with Chemotherapy

According to the latest studies, immunotherapy combined with chemotherapy serves as the first‐line treatment option for advanced NSCLC, regardless of the level of PD‐L1 expression. IMpower130, a multicenter, randomized, open phase III study in April 2015, compared atezolizumab with the combined carboplatin and pemetrexed chemotherapy regimen [122]. The result demonstrated a median OS of 18.6 versus 13.9 months, respectively [122]. In January 2016 and June 2016, KEYNOTE‐189 and KEYNOTE‐407 studies were initiated, both of which suggested that among advanced squamous and nonsquamous NSCLC, pembrolizumab combined with platinum‐based chemotherapy (combined pemetrexed and albumin paclitaxel) significantly improved the prognosis of patients (KEYNOTE‐189: PFS 9.0 vs. 4.9 months and OS 22.0 vs. 10.6 months; KEYNOTE‐407: PFS 8.0 vs. 5.1 months and OS 17.2 vs. 11.6 months) [123, 124]. In addition, camrelizumab, sintilimab, tislelizumab, sugemalimab, toripalimab, pembrolizumab, and serplulimab combined with chemotherapy can be used as a first‐line treatment in advanced driver gene‐positive NSCLC. All of the above ICIs have been approved by US FDA and NMPA, which achieved satisfactory results in clinical practice. IMpower150 pays attention on wild‐type genotype NSCLC patients, whose EGFR or ALK mutations were excluded. Socinski et al. [125] published the result of IMpower150 in 2018, which showed that when compared with the combination of bevacizumab+carboplatin+paclitaxel (BCP group), atezolizumab+bevacizumab+carboplatin+paclitaxel (ABCP group) showed better median PFS (8.4 vs. 6.8 months) and median OS (19.0 vs. 14.7 months). The ABCP group also has better ORR and duration of remission (DoR) (ORR: 63.5 vs. 48.0%; DoR: 9.0 vs. 5.7 months) [125]. The study confirms the synergistic effects of ICIs and antiangiogenic agents. It also demonstrated the ability of bevacizumab to restore cellular immune function by remodeling the TME, opening new paths of combination therapy in advanced, drug‐resistant NSCLC.

#### 3.3.3 Dual ICIs

Previous studies suggested a higher occurrence of irAEs in melanoma patients treated with the combination of nivolumab and ipilimumab compared with monotherapy. On the other hand, dual immunotherapy has shown efficacy in metastatic NSCLC. In the 2023 World Conference on Lung Cancer, CheckMate 227 showed that after 6 years of follow‐up, nivolumab in combination with ipilimumab could improve OS over standard chemotherapy by 3.2 months (HR = 0.73), and the 5‐year OS rates for nivolumab in combination with ipilimumab versus chemotherapy were 24 versus 14% (PD‐L1 ≥ 1%) and 19 versus 7% (PD‐L1 < 1%), respectively [126]. The result of this study is encouraging, as CTLA4 inhibitors plus PD‐1 inhibitors provide durable benefits to patients with advanced lung cancer by promoting T‐cell proliferation and subsequent antitumor immune response.

#### 3.3.4 Ongoing Combination Therapy

The exploration of immunotherapy in advanced NSCLC continues, with ongoing clinical trials EVOKE‐02 (NCT05186974) and GALLANT‐1 (NCT05240131), which are open, multicenter studies on advanced NSCLC. EVOKE‐02 explored the potential of dual ICIs in combination with various chemotherapy regimens in advanced NSCLC, which enrolled 193 patients to study the therapeutic effect of Sacituzumab in combination with pembrolizumab [127]. GB1211, a novel oral Gal‐3 inhibitor, has shown preliminary positive results in GALLANT‐1 for the first‐line management of advanced or metastatic NSCLC in combination with atezolizumab. 100 mg of GB1211 showed a better safety profile with a lower rate of irAEs, along with better efficacy [128]. Meanwhile, TROPION‐LUNG 01 study (NCT04656652) is a global, multicenter, open‐label phase III randomized controlled study comparing the efficacy and safety of dextrastuzumab and docetaxel in driver gene‐positive/negative stage IIIB‐IV NSCLC patients previously treated with chemotherapy and immunotherapy. Result from this study suggests that dextrastuzumab exhibited a statistically significant beneficial PFS in the population, even if enrolled patients are previously treated with immunotherapy and are immune‐resistant [129].

Immunotherapy for advanced NSCLC has been advancing; immunohistochemistry for PD‐L1 expression allows for an initial assessment of whether a patient can benefit from PD‐1/PD‐L1 therapy. Even in patients with negative PD‐L1 expression, data have shown that immunotherapy can provide survival benefits [121]. Existing clinical trials are discovering new therapeutic combinations, studies such as GALLANT‐1, expect to explore the potential of the novel Gal‐3 inhibitor GB1211 (initially used as a treatment for cirrhosis) in advanced NSCLC, providing new approach for the management of advanced NSCLC. With the goal of personalized precision immunotherapy, further development of biomarkers and exploration of combination immunotherapy regimens with ideal safety profiles will be carried out.

### 3.4 Breast Cancer

Breast cancer, as the most common malignancy among women, has always been a hot spot in clinical research. In recent decades, the prognosis of breast cancer patients has been significantly improved by chemotherapy, targeted therapy, and RT. However, in breast cancers with chemotherapy ineffectiveness or drug resistance, especially triple negative breast cancer (TNBC), finding an effective treatment scheme is still a major challenge. Traditionally, breast cancer is regarded as a “cold” tumor, because of its limited T cell infiltration and low TMB. However, recent studies have observed abundant active infiltrating lymphocytes in TNBC, creating opportunities for the application of ICIs. The presence of these lymphocytes not only reflects the antitumor immune response in the TME, but is also related to the prognosis and chemotherapy response of patients [130]. Through microarray‐based analysis of immune related tumor gene expression, researchers found a significant correlation between the immune gene characteristics of TNBC and HER2 positive breast cancer with good clinical results [131, 132]. This discovery has opened up new ideas for the community, bringing new hope to breast cancer patients. Building upon these findings, we systematically compiled pivotal clinical trials evaluating ICIs in TNBC with distinct immune gene signatures (Table 4).

**TABLE 4:** Clinical trials of ICIs for triple negative breast cancer.

Tumor type	Clinical trial name	Registration number	Country	Completion date	Starting date	LOT	Phase	Combination type	Regimens	No. of patients	mPFS (months)	mOS (months)	ORR	Grade 3/4 TRAEs
**Clinical trials with results of ICIs for triple negative breast cancer (TNBC)**														
Advanced TNBC	KEYNOTE‐012	NCT01848834	NA	2020/6/30	2013/5/7	NA	Ib	Single‐agent	Pembrolizumab	32	1.9	11.2	18.5%	15.6%
Locally advanced or metastatic breast cancer	—	NCT01772004	Multiple countries	2019/12/16	2013/1/31	NA	Ib	Single‐agent	Avelumab	168	NA	NA	5.2%	13.7%
ER+/HER2‐advanced breast cancer	KEYNOTE‐028	NCT02054806	Multiple countries	2021/4/30	2014/2/17	NA	Ib	Single‐agent	Pembrolizumab	25	12.0	NA	12.0%	16.0%
Advanced TNBC	KEYNOTE‐086	NCT02447003	Multiple countries	2020/1/31	2015/6/11	NA	II	Single‐agent	Pembrolizumab	170	2.0	9.0	5.3%	12.9%
Metastatic TNBC	KEYNOTE‐119	NCT02555657	Multiple countries	2020/11/10	2015/10/13	NA	III	Single‐agent	Pembrolizumab	312	2.1	10.7	30.0%	20.0%
Stage II/III of breast cancer	I‐SPY2	NCT01042379	United States	2031/12/1	2010/3/1	NA	II	Chemotherapy + ICIs (anti‐PD‐1)	Neoadjuvant chemotherapy + pembrolizumab	250	NA	NA	60.0% (pCR)	NA
Metastatic TNBC	—	NCT01633970	United States	2020/2/26	2012/7/11	First‐line	Ib	Chemotherapy + ICIs (anti‐PD‐L1)	Nab‐paclitaxel + FOLFOX + atezolizumab	33	5.5	14.7	39.4%	73.0%
Untreated metastatic TNBC	—	NCT02425891	Multiple countries	2021/8/31	2015/6/23	First‐line	III	Chemotherapy + ICIs (anti‐PD‐L1)	Nab‐paclitaxel + atezolizumab	451	7.2	21.3	56.0%	15.9%
Early‐stage TNBC	KEYNOTE‐173	NCT02622074	Multiple countries	2019/11/18	2016/1/27	NA	Ib	Chemotherapy + ICIs (anti‐PD‐1)	Anthracycline + taxane‐based chemotherapy + pembrolizumab	60	NA	NA	60.0% (pCR)	10.0%
Untreated locally recurrent inoperable or metastatic TNBC	—	NCT02819518	Multiple countries	2023/10/30	2016/7/27	NA	III	Chemotherapy + ICIs (anti‐PD‐1)	Chemotherapy + pembrolizumab	847	9.7	23.0	40.8%	68.1%
Untreated stage II or III TNBC	KEYNOTE‐522	NCT03036488	Multiple countries	2024/3/22	2017/3/7	NA	III	Chemotherapy + ICIs (anti‐PD‐1)	Neoadjuvant chemotherapy + pembrolizumab	1174	NA	NA	NA	78.0%
Early‐stage TNBC	IMpassion031	NCT03197935	Multiple countries	2022/9/28	2017/7/24	NA	III	Chemotherapy + ICIs (anti‐PD‐L1)	Nab‐pac‐AC + atezolizumab	455	NA	NA	58.0% (pCR)	23.0%
Locally advanced/metastatic TNBC	IMpassion131	NCT03125902	Multiple countries	2023/1/17	2017/8/25	NA	III	Chemotherapy + ICIs (anti‐PD‐L1)	Paclitaxel + atezolizumab	651	6.0	NA	NA	11.0%
Metastatic TNBC	—	NCT03800836	United States	2022/3/16	2018/2/13	NA	Ib	Chemotherapy + ICIs (anti‐PD‐L1)	Lpatasertib + paclitaxel/Nab‐paclitaxel + atezolizuma	317	NA	NA	73.0%	53.0%
TNBC	AGO‐B‐041	NCT03289819	Germany	2021/1/22	2018/3/23	NA	II	Chemotherapy + ICIs (anti‐PD‐1)	Nab‐paclitaxel/epirubicin + cyclophosphamide + pembrolizumab	50	NA	NA	66.0%	64.2%
Metastatic TNBC	ENHANCE‐1	NCT02513472	United States	2021/4/6	2015/8/28	NA	I/II	Targeted therapy (anti‐TLR4) + ICIs (anti‐PD‐1)	Eribulin + pembrolizumab	258	NA	NA	23.4%	NA
BRCA‐mutated metastatic breast cancer	MEDIOLA	NCT02734004	Multiple countries	2021/9/17	2016/3/17	NA	I/II	Targeted therapy (anti‐VEGF) + PARPi + ICIs (anti‐PD‐L1)	Bevacizumab + olaparib + durvalumab	34	8.2	21.5	58.8%	32.0%
Advanced TNBC	—	NCT03394287	China	2020/9/30	2018/1/10	NA	II	Targeted therapy (anti‐VEGFR2) + ICIs (anti‐PD‐1)	Apatinib + camrelizumab	40	3.7 vs. 1.9	NA	63.3 vs. 40.0%	46.7%
Advanced TNBC	LEAP‐005	NCT03797326	United States	2024/10/28	2019/2/12	NA	II	Targeted therapy (anti‐VEGF)+ICIs (anti‐PD‐1)	Lenvatinib + pembrolizumab	31	5.1	11.4	23.0%	52.0%
TNBC	—	NCT04129996	China	2022/9/1	2019/10/1	NA	II	Chemotherapy + Targeted therapy (anti‐VEGF) + ICIs (anti‐PD‐1)	Chemotherapy + angiogenesis inhibitor + camrelizumab	360	13.6	NA	81.3%	NA
Metastatic TNBC	—	NCT02708680	United States	2021/3/10	2016/5/1	NA	I/II	ADC + ICIs (anti‐PD‐1)	Entinostat + atezolizumab	40	1.7	12.3	12.5%	NA
Metastatic TNBC	—	NCT02730130	United States	2022/10/12	2016/6/3	NA	II	RT + ICIs (anti‐PD‐1)	Radiotherapy + pembrolizumab	17	NA	NA	17.6%	23.5%
Advanced/metastatic TNBC	—	NCT02657889	United States	2021/9/17	2016/4/15	NA	II	PARPi + ICIs (anti‐PD‐1)	Niraparib + pembrolizumab	55	2.3	NA	21.0%	40.0%
Advanced TNBC	—	NCT03330405	United States	2023/1/4	2017/10/19	NA	I/II	PARPi + ICIs (anti‐PD‐L1)	Talazoparib + avelumab	223	NA	NA	18.2%	NA
**Ongoing clinical trials of ICIs for triple negative breast cancer (TNBC)**														
TNBC	—	NCT03310957	Multiple countries	2018/2/27	Ongoing	NA	I/II	ADC + ICIs (anti‐PD‐1)	SGN‐LIV1A + pembrolizumab	186				
Metastatic TNBC	—	NCT03742102	Multiple countries	2018/12/21	Ongoing	NA	I/II	ADC + ICIs (anti‐PD‐L1)	Trastuzumab deruxtecan + durvalumab	243				
Refractory or Relapsed (R/R) TNBC	—	NCT03752723	Korea	2019/3/27	Ongoing	NA	I/II	Recombinant human interleukin‐7 (IL‐7) + ICIs (anti‐PD‐1)	GX‐I7 + pembrolizumab	84				

The trials in the table are arranged in the order of their appearances in the main text. For trials not explicitly discussed in the text, they are ordered chronologically by trial initiation dates (ascending order).

*Data sources*: ClinicalTrials.gov (clinicaltrials.gov), PubMed, Wanfang Data (https://www.wanfangdata.com.cn/), ESMO (European Society for Medical Oncology), and ASCO (American Society of Clinical Oncology—ASCO).

Abbreviations: anti‐TLR4, anti‐Toll‐like receptor 4; LOT, lines of therapy; Nab‐pac‐AC, Nanoparticle Albumin‐Bound Paclitaxel (Nab‐paclitaxel) combined with Adriamycin (Doxorubicin) and Cyclophosphamide; PARPi, poly (ADP‐ribose) polymerase inhibitor; TNBC, triple negative breast cancer.

#### 3.4.1 ICIs Monotherapy

In 2016, Nanda et al. [133] published a phase Ib trial on the effectiveness of single‐agent pembrolizumab for advanced TNBC. Among the 32 patients with advanced TNBC included in the study, the ORR was 18.5%, and the median time to response was 17.9 weeks (range, 7.3–32.4 weeks) [133]. This provided preliminary evidence of the effectiveness and safety of pembrolizumab in patients with TNBC. Another phase Ib clinical study evaluated the efficacy of another ICI, atezolizumab, in TNBC. This study assessed atezolizumab in 116 patients with metastatic TNBC, with a median PFS of 1.4 months and an ORR of 10% among all TNBC patients. In PD‐1 positive patients, the ORR increased to 12%. Of all patients receiving treatment, 73 (63%) experienced irAEs, most of these events were grade 1 to 2 (58 cases, 79%), only three patients (3%) discontinued treatment due to intolerable irAEs, suggesting that monotherapy with atezolizumab is well tolerated and can offer lasting clinical benefits to patients with stable or responding metastatic TNBC [134].

#### 3.4.2 Combination with Chemotherapy

With the ongoing progress of research, combination therapy with ICIs is gradually demonstrating advantages in breast cancer. Based on monotherapy with atezolizumab, Schmid et al. [135] conducted IMpassion130. This study is a global, multicenter, randomized phase III clinical trial. It mainly studies atezolizumab in combination with nab‐paclitaxel as first‐line management for locally advanced or metastatic TNBC, with the primary endpoints being PFS and OS. In this trial, patients were assigned randomly (in a 1:1 ratio) to receive atezolizumab+nab‐paclitaxel or placebo+nab‐paclitaxel. The median PFS was 7.2 months for the atezolizumab plus nab‐paclitaxel group, compared with 5.5 months for the placebo plus Nab‐paclitaxel group (HR = 0.80; 95% CI: 0.69–0.92; *p* = 0.002) [135]. The median OS improved from 15.5 months in the placebo group to 25.0 months in the atezolizumab plus Nab‐paclitaxel group, without new irAEs being reported [135]. Overall, the atezolizumab plus Nab‐paclitaxel regimen extends PFS for untreated metastatic TNBC patients and demonstrates reliable safety profile [135]. In 2019, US FDA approved atezolizumab in combination with nab‐paclitaxel for the first‐line treatment of unresectable locally advanced or metastatic PD‐L1 positive TNBC, making it the first immunotherapy regimen for breast cancer. In 2021, atezolizumab was officially incorporated into the NCCN guidelines for breast cancer, specifically for the management of locally advanced or metastatic TNBC, marking a significant milestone in the field of immunotherapy for breast cancer [136].

In addition to their strong performance in advanced TNBC, recent studies have shown that combining pembrolizumab to neoadjuvant chemotherapy significantly increases the pCR rate in early‐stage TNBC patients. This phase III trial enrolled previously untreated patients with stage II and III TNBC, randomly assigning them in a 2:1 ratio to either the pembrolizumab plus chemotherapy group (784 patients, receiving four cycles of pembrolizumab with paclitaxel and carboplatin every 3 weeks) or the placebo plus chemotherapy group (390 patients, receiving placebo with paclitaxel and carboplatin every 3 weeks) [137]. Among the first 602 randomly assigned patients, the pCR rate in the pembrolizumab plus chemotherapy group was 64.8% (260 of 401 patients), compared with 51.2% (103 out of 201 patients) for the placebo group, which is statistically significant [137]. These results demonstrate that pembrolizumab significantly improves the pCR rate in patients receiving neoadjuvant chemotherapy with platinum‐based agents, including those with low PD‐L1 expression, who also experienced notable benefits [137]. This result is also consistent with previous phase II study results [138]. Findings from several large‐scale prospective cohort trials show that ICIs benefit a wide range of populations in TNBC (including early‐stage and advanced, unresectable tumors), and they demonstrate reliable safety profile, greatly improving treatment for TNBC.

#### 3.4.3 Combination with Targeted Therapy

In addition to TNBC, researchers are also exploring the efficacy of ICIs in patients with HER2‐positive breast cancer. IMpassion050 evaluated the effectiveness and safety of atezolizumab combined with trastuzumab, pertuzumab, and preoperative neoadjuvant chemotherapy in early stage high‐risk HER2‐positive breast cancer patients. Unfortunately, compared with standard dual‐ICIs neoadjuvant therapy, the immunotherapy combination did not improve the pCR in the placebo and atezolizumab groups (62.7 and 62.4%, with a difference of −0.33%; 95% CI, −9.2 to 8.6; *p* = 0.955) [139]. At the same time, KATE2 (NCT02924883) assessed the effectiveness and safety of T‐DM1 plus atezolizumab in locally advanced or metastatic HER2‐positive breast cancer patients previously managed with trastuzumab and taxane‐based therapies, the results indicated that the addition of atezolizumab to the T‐DM1 regimen did not yield a significant PFS benefit in the intention‐to‐treat (ITT) population. The median PFS was 8.2 months for patients receiving atezolizumab, compared with 6.8 months for those receiving placebo [140]. Neo‐PATH, a single‐arm, nonrandomized trial, investigated preoperative atezolizumab combined with docetaxel, trastuzumab, and pertuzumab in stage II or III HER2‐positive breast cancer patients. Research findings demonstrated an overall pCR rate of 61%, with a significantly higher pCR rate in PD‐1 positive patients compared with negative patients (100 vs. 53%), suggesting beneficial pCR rate with combination therapy of ICIs, anti‐HER2 targeted therapy and chemotherapy in HER2‐positive breast cancer patients [141]. Future research is needed to further explore ICIs therapy in HER2‐positive breast cancer.

#### 3.4.4 Emerging Combination Therapies

A phase Ib clinical study (KEYNOTE‐028, NCT02054806) found that pembrolizumab monotherapy showed a modest but sustained overall response in some patients with previously treated, advanced, PD‐L1 positive, ER+/HER2‐breast cancer, with an ORR of 12% (95% CI, 2.5–31.2%), and a median DoR of 12.0 months [142]. This established a basis for ICIs therapy in HR+ breast cancer patients. Wu et al. [143] presented successful outcomes in two patients receiving immunotherapy and endocrine therapy. Both patients with metastatic HR+ breast cancer were treated with pembrolizumab in combination with either letrozole or tamoxifen, and both had a PFS of over 21 months, indicating improved survival outcomes with the combination of ICIs with endocrine therapy in metastatic HR+ breast cancer patients [143]. Yuan et al. [144] reported on the efficacy of the combination of palbociclib, pembrolizumab and letrozole in stage IV HR+ HER2‐metastatic breast cancer, among the 23 enrolled patients who received this combination as first‐line treatment, the ORR was 56% (31% achieved CR and 25% achieved PR).

The combination of ICIs with other treatment modalities has improved survival outcomes for some breast cancer patients. As research progresses, immunotherapy for breast cancer is advancing, and its safety has been confirmed. Future developments of ICIs therapy in the field of breast cancer will mainly focus on their combination with chemotherapy, targeted therapies and novel targets such as BRCA, PI3K, AKT, mTOR, MEK, and ADCs [145, 146, 147, 148, 149].

### 3.5 Hepatocellular carcinoma

Liver cancer was identified as the 6th most prevalent malignancy and is recognized as the 3rd highest cause of death due to cancer globally in 2022 [1]. HCC represents the majority of the histological type of liver cancer, constituting for at least 90% of cases [150]. A significant proportion of individuals with HCC are diagnosed in later stages of the disease, with majority eventually requiring systemic therapy. Over the past few years, there has been a notable advancement in the field of systemic therapy, particularly with the use of ICIs, which have shown considerable potential in liver cancer. We conducted an in‐depth review of the application of ICIs in advanced HCC (aHCC) and summarized clinical trial data with available results according to the types of ICIs and their applications, as well as ongoing clinical trials in Table 5.

**TABLE 5:** Clinical trials of ICIs for hepatocellular carcinoma.

Tumor type	Clinical trial name	Registration number	Country	Completion date	Starting date	LOT	Phase	Combination type	Regimens	No. of patients	mPFS (months)	mOS (months)	ORR	Grade 3/4 TRAEs
**Clinical trials with results of ICIs for hepatocellular carcinoma (HCC)**														
HCC	SHARP	NCT00105443	Multiple countries	2008/11/1	2005/3/1	First‐line	III	Single‐agent (TKI)	Sorafenib	299	5.5	10.7	2.0%	18.0%
HCC	—	NCT01008358	Spain	2012/5/1	2008/12/1	—	II	Single‐agent	Tremelimumab	20	6.5	8.2	17.6%	45.0%
HCC	Checkmate040	NCT01658878	Multiple countries	2024/11/12	2012/10/30	Second‐line	I/II	Single‐agent	Nivolumab (in the dose‐expansion phase)	214	4.1	15.6	20.0%	19.0%
HCC	Checkmate459	NCT02576509	Multiple countries	2024/2/7	2015/12/7	Second‐line	III	Single‐agent	Nivolumab vs. sorafenib	371 vs. 372	3.7 vs. 3.8	16.4 vs. 14.7	15 vs. 7%	22.0 vs. 49.0%
HCC	KEYNOTE‐224 (MK‐3475‐224)	NCT02702414	Multiple countries	2023/9/29	2016/5/31	Second‐line	II	Single‐agent	Pembrolizumb (previously treated with sorafenib)	104	4.9	12.9	17.0%	24.0%
									Pembrolizumb (previously untreated)	51	4	17	16.0%	16.0%
HCC	KEYNOTE‐240(MK‐3475‐240)	NCT02702401	Multiple countries	2021/9/22	2016/5/26	Second‐line	III	Single‐agent	Pembrolizumb vs. placebo	278 vs. 135	3.0 vs. 2.8	13.9 vs. 10.6	18.3 vs. 4.4%	52.7 vs. 46.3%
									Pembrolizumb vs. placebo	Asian: 107 vs. 50	2.8 vs. 1.4	13.8 vs. 8.3	20.6 vs. 2.0%	13.1 vs. 4.0%
HCC	KEYNOTE‐394(MK‐3475‐394)	NCT03062358	Multiple countries	2024/10/15	2017/4/27	Second‐line	III	Single‐agent	Pembrolizumb vs. placebo	300 vs. 153	2.6 vs. 2.3	14.6 vs. 13.0	12.7 vs. 1.3%	14.3 vs. 5.9%
HCC	—	NCT02989922	China	2020/3/3	2016/11/15	Second‐line	II	Single‐agent	Camrelizumab	217	2.1	13.8	14.7%	22.0%
HCC	RATIONALE‐208	NCT03419897	Multiple countries	2022/7/6	2018/4/9	Second‐line	II	Single‐agent	Tislelizumab	249	2.7	13.2	13.3%	15.0%
HCC	RATIONALE‐301	NCT03412773	Multiple countries	2023/12/14	2017/12/28	First‐line	III	Single‐agent	Tislelizumab vs. sorafenib	342 vs. 332	2.1 vs. 3.4	15.9 vs. 14.1	14.3 vs. 5.4%	22.2 vs. 53.4%
HCC	—	ChiCTR2000037655	China	2022/4/23	2020/9/1	NA	II	Single‐agent	Sintilimab vs. active surveillance group	99 vs. 99	NA	27.7 vs. 15.5	NA	12.4%
HCC	G030140	NCT02715531	Multiple countries	2021/5/31	2016/4/6	First‐line	Ib	Targeted therapy (anti‐VEGF) + ICI (anti‐PD‐L1)	Bevacizumab + atezolizumab	104	7.3	17.1	36.0%	39.0%
HCC	IMbrave150	NCT03434379	Multiple countries	2022/11/17	2018/3/15	First‐line	III	Targeted therapy (anti‐VEGF) + ICI (anti‐PD‐L1)	Bevacizumab + atezolizumab	336	6.9	19.2	29.8%	43.0%
HCC	ORIENT‐32	NCT03794440	China	2022/12/1	2019/2/11	First‐line	II/III	Targeted therapy (anti‐VEGF) + ICI (anti‐PD‐1)	IBI305 + sintilimab vs. sorafenib	380 vs. 191	4.6 vs. 2.8	Not reached vs. 10.4	20.3 vs. 4.1%	33.7 vs. 35.7%
HCC	—	NCT03006926	Multiple countries	2022/11/22	2017/2/13	NA	Ib	Targeted therapy (TKI) + ICI (anti‐PD‐1)	Lenvatinib + pembrolizumab	104	8.6	22	46.0%	67.0%
HCC	COSMIC‐312	NCT03755791	Multiple countries	2024/12/1	2018/6/10	First‐line	III	Targeted therapy (TKI) + ICI (anti‐PD‐L1)	Cabozantinib + atezolizumab vs. sorafenib	432 vs. 217	6.8 vs. 4.2	15.4 vs. 15.5	11 vs. 4%	18 vs. 8%
HCC	MK‐7902‐002(E7080‐G000‐311/​LEAP‐002)	NCT03713593	Multiple countries	2024/9/24	2018/12/31	First‐line	III	Targeted therapy (TKI) + ICI (anti‐PD‐1)	Lenvatinib + pembrolizumab vs. lenvatinib + placebo	395 vs. 399	8.2 vs. 8.0	21.2 vs. 19.0	26.1 vs. 17.5%	62.5 vs. 57.5%
HCC	CARES‐310	NCT03764293	Multiple countries	2023/6/14	2019/6/10	First‐line	III	Targeted therapy (TKI) + ICI (anti‐PD‐1)	Apatinib + camrelizumab vs. sorafenib	272 vs. 271	5.6 vs. 3.7	22.1 vs. 15.2	25.4 vs. 5.9%	24 vs. 6%
HCC	BGB‐A317‐211	NCT04401800	China	2024/2/18	2020/9/4	First‐line	II	Targeted therapy (TKI) + ICI (anti‐PD‐1)	Lenvatinib + tislelizumab	64	8.2	Not reached	38.7%	28.1%
HCC	—	NCT03519997	United States	2025/4/1	2018/4/26	NA	II	Targeted therapy (anti‐phosphatidylserine) + ICI (anti‐PD‐1)	Bavituximab + pembrolizumab	35	6.3	NA	32.1%	14.3%
HCC	—	NCT03893695	Taiwan	2022/9/27	2019/5/25	NA	Ib/II	Targeted therapy (anti‐ALK‐1) + ICI (anti‐PD‐1)	GT90001 + nivolumab	20	2.81	NA	30.0%	30.0%
HCC	EMERALD‐1	NCT03778957	Multiple countries	2026/8/31	2018/11/30	NA	III	TACE + targeted therapy + ICI (anti‐PD‐1)	TACE + durvalumab vs. TACE + durvalumab+bevacizumab vs. TACE +placebo	616	10.0 vs. 15.0 vs. 8.2	NA	41 vs. 43.6 vs. 29.6%	15.1 vs. 32.5 vs. 13.5%
HCC	START‐FIT	NCT03817736	Hong Kong	2023/7/26	2019/3/1	NA	II	TACE+SBRT + ICI (anti‐PD‐L1)	TACE + SBRT + avelumab	33	20.7	30.3	67.0%	33.0%
HCC	—	NCT04599790	China	2022/10/31	2020/10/1	NA	II	TACE + targeted therapy +ICI (anti‐PD‐1)	TACE + lenvatinib + sintilimab	30	8	18.4	60.0%	40.0%
HCC	—	CHiCTR2000039508	China	2023/10/31	2020/11/1	NA	IV	TACE + targeted therapy +ICI (anti‐PD‐1)	TACE + TKI + camrelizumab	87	Not reached	10.5	71.3%	67.8%
HCC	PLATIC	NCT04814043	China	2023/12/30	2021/4/20	NA	II	TACE–HAIC + targeted therapy + ICI (anti‐PD‐1)	TACE–HAIC + targeted + lenvatinib + sintilimab	57	14.3	NA	80.7%‐mRECIST; 42.1%‐RECIST 1.1	64.9%
HCC	—	NCT01853618	United States	2013/5/2	2017/6/7	NA	II	RFA + ICI (anti‐CTLA‐4)	RFA + tremelimumab	32	7.4	12.3	26.3%	NA
HCC	CA209‐678	NCT03033446	Singapore	2024/12/31	2016/12/20	NA	II	RT+ICI (anti‐PD‐1)	RT + nivolumab	36	3.6	16.9	30.6%	6.0%
HCC	HCRNGI15‐225	NCT03099564	United States	2023/6/1	2017/3/28	NA	I	RT + ICI (anti‐PD‐1)	RT + pembrolizumab	27	9.9	27.3	30.8%	48.1%
HCC	—	NCT04044313	China	2022/2/28	2019/8/1	NA	II	HAIC + targeted therapy + ICI (anti‐PD‐1)	HAIC + lenvatinib + toripalimab	36	10.4	17.9	63.9%	72.2%
HCC	TRIPLET	NCT04191889	China	2025/12/31	2020/4/13	NA	II	HAIC + targeted therapy + ICI (anti‐PD‐1)	HAIC + apatinib + camrelizumab	35	10.4	Not reached	77.1%	37.1%
HCC	—	NCT02519348	Multiple countries	2025/3/31	2015/10/19	NA	I/II	Dual‐ICIs (anti‐PD‐L1 + anti‐CTLA‐4)	Durvalumab + tremelimumab (330 mg) vs. durvalumab vs. tremelimumab vs. durvalumab + tremelimumab (75 mg)	75 vs. 104 vs. 69 vs. 84	2.2 vs. 2.1 vs. 2.7 vs. 1.9	18.7 vs. 13.6 vs. 15.1 vs. 11.3	24.0 vs. 10.6 vs. 7.2 vs. 9.5%	37.8 vs. 20.8 vs. 43.5 vs. 24.4%
HCC	—	NCT03222076	United States	2022/9/14	2017/9/28	NA	II	Dual‐ICIs (anti‐PD‐1 + anti‐CTLA‐4)	Nivolumab + ipilimumab vs. nivolumab	14 vs. 13	19.5 vs. 9.4	NA	NA	43.0 vs. 23.0%
HCC	HIMALAYA	NCT03298451	Multiple countries	2026/8/27	2017/10/11	First‐line	III	Dual‐ICIs (anti‐PD‐L1 + anti‐CTLA‐4)	Durvalumab + tremelimumab	393	3.8	16.4	20.1%	26.0%
									Durvalumab + tremelimumab	Asian: 156	NA	16.5	28.2%	19.9%
HCC	DUBHE‐H‐308	NCT05976568	China	2027/9/1	2023/9/1	First‐line	Ib/II	ICIs (anti‐PD‐1/CTLA‐4) + targeted therapy (anti‐VEGF)/chemotherapy	QL1706 + bevacizumab + chemotherapy vs. QL1706 + bevacizumab vs. QL1706 + chemotherapy vs. sintilimab + bevacizumab	31 vs. 30 vs. 30 vs. 29	Not reached vs. 8.1 vs. 7.0 vs. 5.9	NA	35.5 vs. 36.7 vs. 36.7 vs. 13.8%	46.7 vs. 50.0 vs. 46.7 vs. 37.9%
**Ongoing clinical trials of ICIs for hepatocellular carcinoma (HCC)**														
HCC	—	NCT03821935	Multiple countries	Ongoing	2019/2/21	—	I	Anti‐GARP/TGFβ_1_ + ICI (anti‐PD‐1)	Livmoniplimab + budigalimab	362	NA	NA	NA	NA
HCC	LIVIGNO‐1	NCT05822752	Multiple countries	Ongoing	2023/9/21	—	II	Anti‐GARP/TGFβ_1_ + ICI (anti‐PD‐1)	Livmoniplimab + budigalimab	130	NA	NA	NA	NA
HCC	ZENOBIA	NCT06698250	United States	Ongoing	2024/12/9	—	II	Targeted therapy (TKI) + ICIs (anti‐PD‐L1 + anti‐CTLA‐4)	Zanzalintinib + durvalumab + tremelimumab	40	NA	NA	NA	NA
HCC	—	NCT06294548	United States	Ongoing	2025/2/1	—	I/II	Targeted therapy (anti‐EZH1/2) + ICIs (anti‐PD‐L1) + targeted therapy (anti‐VEGF)	Valemetostat tosylate (DS‐3201b) + atezolizumab + bevacizumab	45	NA	NA	NA	NA
HCC	TPST‐1120‐301	NCT06680258	—	Ongoing	2025/3/29	First‐Line	III	Targeted therapy (anti‐PPARα) + ICIs (anti‐PD‐L1) + targeted therapy (anti‐VEGF)	TPST‐1120 + atezolizumab + bevacizumab	740	NA	NA	NA	NA
HCC with portal vein tumor thrombosis	PATENCY2	NCT06669377	China	Ongoing	2024/1/1	—	Observational	TACE + ICIs + targeted therapy	TACE + ICIs + targeted therapy	444	NA	NA	NA	NA
HCC	—	NCT06375317	—	Ongoing	2024/4/30	—	II	HAIC + ICIs + targeted therapy	HAIC + adebrelimab + regorafenib	42	NA	NA	NA	NA
HCC	—	NCT06530784	China	Ongoing	2024/7/31	Second‐line	II	Cryoablation + ICIs + targeted therapy	Cryoablation + anti‐PD‐1 + bevacizumab	36	NA	NA	NA	NA
HCC	SL‐B2024‐061	NCT06313190	China	Ongoing	2024/4/5	—	II	SBRT + ICIs	SBRT + sintilimab	140	NA	NA	NA	NA
HCC	HSBRT2401	NCT06261125	China	Ongoing	2024/3/10	—	II	SBRT + ICIs + targeted therapy	SBRT + adebrelimab + lenvatinib	60	NA	NA	NA	NA
											NA	NA	NA	NA
HCC	CheckMate 9DW	NCT04039607	Multiple countries	Ongoing	2019/9/30	First‐Line	III	Dual‐ICIs (anti‐PD‐1+anti‐CTLA‐4)	Nivolumab + ipilimumab	732	NA	NA	NA	NA
HCC	SHR‐8068‐301	NCT06618664	China	Ongoing	2024/10/28	First‐Line	III	Dual‐ICIs (anti‐PD‐L1 + anti‐CTLA‐4) + targeted therapy (anti‐VEGF)	Adebrelimab + SHR‐8068 + bevacizumab	560	NA	NA	NA	NA
HCC	ZG005‐005	NCT06558227	China	Ongoing	2024/10/24	First‐Line	II	Anti‐PD‐1/TIGIT +targeted therapy (anti‐VEGF)	ZG005 + bevacizumab	90	NA	NA	NA	NA
HCC	—	NCT04251117	United States, New Zealand	Ongoing	2020/3/1	—	I/IIa	DNA vaccine + plasmid encoded IL‐12 + ICIs (anti‐PD‐1)	GNOS‐PV02 + INO‐9012 + pembrolizumab	36	NA	NA	NA	NA
HCC	—	NCT05155189	China	Ongoing	2021/12/9	—	I	CAR‐T cell injection + ICIs	C‐CAR031 + ICIs	44	NA	NA	NA	NA
HCC	CHS‐388‐202	NCT06679985	—	Ongoing	2024/11/1	—	II	Immunotherapy (anti‐IL‐27) + ICIs (anti‐PD‐1) + targeted therapy (anti‐VEGF)	Casdozokitug + toripalimab + bevacizumab	72	NA	NA	NA	NA
HCC	RZ‐001‐102	NCT06695026	Korea	Ongoing	2024/12/1	—	I/II	Gene therapy + anti‐virus + ICIs (anti‐PD‐L1)/targeted therapy (anti‐VEGF)	RZ‐001 + valganciclovir + atezolizumab/bevacizumab	45	NA	NA	NA	NA

The trials in the table are arranged in the order of their appearances in the main text. For trials not explicitly discussed in the text, they are ordered chronologically by trial initiation dates (ascending order).

*Data sources*: ClinicalTrials.gov (clinicaltrials.gov), PubMed, Wanfang Data (https://www.wanfangdata.com.cn/), ESMO (European Society for Medical Oncology), and ASCO (American Society of Clinical Oncology—ASCO).

Abbreviations: anti‐EZH1/2, antienhancer of Zeste homolog 1/2; anti‐GARP/TGFβ, antiglycoprotein A repetitions predominant/transforming growth factor beta; anti‐PPARα, antiperoxisome proliferator‐activated receptor alpha; CAR‐T cell, chimeric antigen receptor T cell; HAIC, hepatic artery infusion chemotherapy; HCC, hepatocellular carcinoma; PARPi, poly (ADP‐ribose) polymerase inhibitor; RFA, radiofrequency ablation; SBRT, stereotactic body radiation therapy; TACE, transarterial chemoembolization.

#### 3.5.1 ICIs Monotherapy

To date, clinical studies utilizing ICIs as monotherapy have demonstrated promising outcomes, which are instrumental in the management of HCC. Notably, nivolumab and pembrolizumab received US FDA approval for the second‐line treatment of advanced liver cancer.

Tremelimumab is a fully humanized monoclonal antibody targeting CTLA‐4 [25]. The clinical trial NCT01008358 was the first to investigate tremelimumab in HCC patients with hepatitis C virus (HCV) infection. This trial analyzed 20 HCC patients with HCV, resulting in a median PFS of 6.48 months and a median OS of 8.2 months [151]. With the initial use of tremelimumab in HCC, the disease control rate and PFS may provide benefits compared with the results observed in prospective trials of sorafenib [152]. Furthermore, there were indications of potential antiviral effects against HCV with tremelimumab [152].

Nivolumab, a PD‐1 inhibitor also known as “Opdivo,” was approved by US FDA in 2017 for use in the second‐line treatment of aHCC [153]. The multicenter Checkmate 040 played a crucial role in this advancement. The trial's findings showed an ORR of 20% and a mPFS of 4.1 months in the dose‐expansion group of 214 patient, while the dose‐escalation group had an ORR of 15% and a median PFS of 3.4 months [154]. The median OS was 15.6 months in the dose‐expansion group and 15 months in the dose‐escalation group, both superior to sorafenib monotherapy as observed in the SHARP trial [152]. Kudo et al. [155] underwent a deeper analysis of Checkmate 040, suggesting that nivolumab could be an option for aHCC patients with Child‐Pugh B classification. Sangro et al. [156] identified PD‐L1 expression levels as a potential marker for nivolumab efficacy, with at least 1% associated with improved median OS in their analysis of Checkmate 040. The result of Checkmate 459 was presented in The European Society for Medical Oncology (ESMO) in 2019, further confirming the therapeutic value of Nivolumab in aHCC. Yau et al. [157] reported the phase III trial Checkmate 459 in *The Lancet* in 2022, which compared nivolumab with sorafenib in unresectable HCC (uHCC) patients, with nivolumab showing superior OS and ORR (mOS: 16.4 vs. 14.7 months, ORR: 15 vs. 7%). Nivolumab holds its place as a pioneering ICI for aHCC, offering new treatment options for patients who are not candidates for targeted therapies.

Pembrolizumab, also a PD‐1 inhibitor commonly referred to as “K” drug, emerged as another strong contender in the management of advanced liver cancer when it was approved by US FDA in 2018 for second‐line therapy in aHCC. KEYNOTE‐240 was designed to assess the efficacy and safety of pembrolizumab following sorafenib for aHCC patients. While KEYNOTE‐240 did not achieve the predefined statistical thresholds of OS and PFS, it did record a marked improvement in ORR [158]. The subsequent publication by Kudo et al. [159] of the Asian subgroup analysis from KEYNOTE‐240 revealed a more pronounced beneficial trend in Asian patients treated with pembrolizumab. KEYNOTE‐394 (NCT03062358), a phase III RCT, evaluated pembrolizumab as a second‐line therapy for aHCC in Asia, showing significant improvements in OS, PFS, and ORR over placebo (mOS: 14.6 vs. 13.0, mPFS: 2.6 vs. 2.3, ORR: 12.7 vs. 1.3%), along with a favorable safety profile [160]. The collective data from the KEYNOTE series of trials present new therapeutic options for second‐line treatment of advanced liver cancer, with particularly notable clinical benefits observed in Asian populations.

Camrelizumab (SHR‐1210), a PD‐1 inhibitor, showed promising outcomes for aHCC patients in monotherapy from a phase II trial in China (NCT02989922). After a median follow‐up period of 12.5 months, 32 out of 217 camrelizumab patients (14.7%) experienced objective responses, and the survival rate at 6 months reached 74.4% [161]. NMPA approved camrelizumab in the treatment of aHCC in March 2020 base on the above results [162].

Tislelizumab, an IgG4 PD‐1 inhibitor engineered by BeiGene, gained NMPA approval in China for the management of aHCC in June 2021 for use beyond the second‐line and in January 2024 for use in first‐line therapy, these approvals were supported by the outcomes of the RATIONALE 208 and RATIONALE‐301 studies, respectively. RATIONALE 208, a phase II single‐arm global trial, included aHCC patients who are previously treated with systemic therapies. Tislelizumab yielded an overall ORR of 13.3% (per RECIST v1.1), median OS of 13.2 months, mPFS of 2.7 months and a incidence of grade 3+ irAEs of 14.5% [163]. The phase III trial RATIONALE‐301 subsequently evaluated tislelizumab's potential as a first‐line treatment for uHCC. It reported a median OS of 15.9 months for the tislelizumab group versus 14.1 months for the sorafenib group, though the OS benefit of tislelizumab was not statistically significant [164]. Nevertheless, the Kaplan–Meier survival analysis revealed a deferred effect of tislelizumab, with the OS curves for the tislelizumab and sorafenib groups progressively diverging over time, suggesting a potential for tislelizumab in conferring long‐term survival benefits [164]. Additionally, the tislelizumab group exhibited a higher ORR (14.3 compared with 5.4%) and a better safety profile, with a fewer occurrence of grade 3 or higher irAEs [164].

In summary, ICIs offer new options and a solid foundation for first‐line and second‐line immunotherapy in patients with aHCC. However, the efficacy of monotherapy is currently limited, indicating further evaluation. There is a relative scarcity of data on systemic therapy options for HCC patients following ICIs therapy. Recently, Chan et al. [165] found the efficacy of cabozantinib in patients who had previously received ICIs therapy, which fills this gap in research.

#### 3.5.2 Combination with Targeted Therapy

US FDA approved the use of atezolizumab, which targets PD‐L1, in combination with bevacizumab, a humanized monoclonal antibody against vascular endothelial growth factor, for the first‐line therapy of liver cancer on May 29, 2020. This approval was largely based on the positive outcomes of a phase III clinical trial known as IMbrave150. This trial revealed that the combination of atezolizumab and bevacizumab (with a median OS of 19.2 months and median PFS of 6.8 months) outperformed sorafenib, which significantly improved the survival rates of patients with HCC. Coupled with manageable irAEs, the atezolizumab and bevacizumab combination has become the first‐line treatment for HCC that appears to be more effective than the standard therapy since sorafenib in nearly a decade [166]. Additionally, Zhu et al. [167] identified alpha‐fetoprotein as a potential surrogate biomarker in the atezolizumab and bevacizumab treatment regimen for HCC.

Sintilimab combined with IBI305, a bevacizumab biosimilar, demonstrated obvious clinical benefits and acceptable safety profile in the first‐line treatment of aHCC in Chinese patients. These benefits were observed in the ORIENT‐32 multicenter clinical trial, which focused on aHCC patients with HBV infection. The trial indicated that the combination of sintilimab and IBI305 provided substantial improvements in OS and PFS over sorafenib, potentially offering new therapeutic approaches for this patient group [168]. Interestingly, Li et al. [169] analyzed ascites from HCC patients treated with sintilimab and bevacizumab and discovered that IFN signaling could act as a predictive biomarker for immunotherapy. They also suggested that the use of IFN inducers in combination with ICIs might be advantageous for patients with HCC [169].

The combination of ICIs with tyrosine kinase inhibitors (TKI) is emerging as a noteworthy therapeutic strategy for aHCC. The COSMIC‐312 clinical trial assessed the efficacy of cabozantinib (TKI) combined with atezolizumab for aHCC. The findings indicated that while the combination therapy enhanced PFS compared with sorafenib, it did not improve OS significantly and was linked to a higher incidence of irAEs [170]. The phase III LEAP‐002 clinical study subsequently revealed a more pronounced benefit trend for lenvatinib combined with pembrolizumab in the first‐line management of aHCC among the Asian population [171]. In 2023, the CARES‐310 clinical trial by Qin et al. [172] reported its results in *The Lancet*. This phase III clinical trial demonstrated that compared with sorafenib, the combination of camrelizumab and rivoceranib (apatinib) significantly extended median OS (22.1 vs. 15.2 months) and improved median PFS (5.6 vs. 3.7 months) [172]. This marks the first clinical trial to observe positive endpoints for both PFS and OS with the first‐line application of ICIs combined with TKIs in HCC. Furthermore, follow‐up data from the CARES‐310 trial presented at the 2024 ASCO annual meeting indicated that the median OS reached 23.8 months in combination therapy group, which is the longest median survival time reported to date in phase III clinical studies for first‐line treatment of aHCC [173]. Based on the results, the combination therapy of camrelizumab and apatinib is now an approved first‐line treatment for uHCC in China [172].

The integration of targeted therapies with ICIs in HCC is advancing with continuous innovation (see Table 5 for details). Preliminary observations from clinical trials NCT03519997 and NCT03893695 have noted positive therapeutic effects of the combination of the antiphosphatidylserine antibody (bavituximab) and the antimesenchymal–epithelial transition factor receptor tyrosine kinase 1 antibody (GT90001) with ICIs in HCC [174, 175]. Moreover, novel targeted therapy agents such as valemetostat tosylate (targeting enhancer of zeste homolog 1/2 histone lysine N‐methyltransferase) and TPST‐1120 (a peroxisome proliferator‐activated receptor alpha antagonist) are actively being explored in combination with ICIs for HCC.

#### 3.5.3 Combined with Local Treatments

Transarterial chemoembolization (TACE) can trigger immunomodulatory effects that may enhance the therapeutic efficacy of ICIs [176]. Clinical trials combining TACE with ICIs have also demonstrated enhanced efficacy. At the 2024 ASCO, Lencioni et al. [177] reported the latest results of the EMERALD‐1 clinical trial, showing that the median PFS in patients with uHCC treated with TACE plus durvalumab and bevacizumab was 15 months, significantly longer than the median PFS of the TACE group (8.2 months). This trial is the first to exhibit a statistically significant improvement in PFS among uHCC treated with TACE combined with a targeted‐immunotherapy regimen. At the 2024 ASCO annual meeting, Yuan et al. [178] announced the results of the PLATIC clinical trial, which investigated PD‐1 inhibitors combined with lenvatinib and TACE–hepatic arterial infusion chemotherapy (HAIC) as a conversion treatment for initially unresectable HCC patients. After three cycles of treatment, it achieved a preoperative conversion rate of 77.2%, an ORR of 42.1% (per RECIST 1.1) and a median PFS of 14.3 months [178]. However, the high occurrence of grade 3/4 irAEs (64.9%) cannot be ignored [178].

Radiofrequency ablation (RFA) is a technique that uses heat generated by high‐frequency electrical currents to cause tumor tissue necrosis [179]. Relevant animal trials showed that in situ tumor destruction techniques like RFA can stimulate antitumor immunity by causing release of antigens [180]. A phase II clinical study (NCT01853618) assessed the effectiveness and safety of RFA combined with nivolumab for HCC, showing a median PFS of7.4 months and median OS of 12.3 months, demonstrating the therapeutic potential of this combination therapy in patients with aHCC.

RT destroys tumor cells and inhibits their proliferation through high‐energy radiation. Relevant preclinical trials have shown that the combination of ICIs and RT significantly improved tumor growth delay and antitumor effects in mouse models [181]. Some early clinical trials have found that ICIs combined with RT shows promising efficacy [182, 183]. The combination has shown positive prospects in preclinical and early clinical trials, but its long‐term effects and optimal treatment strategies still require further research for clarification.

HAIC is a treatment method that directly infuses chemotherapy drugs into the hepatic artery. The NCT04044313 study investigated the effectiveness and safety of HAIC combined with durvalumab and bevacizumab in patients with aHCC, with the combined treatment group's median PFS of 10.4 months and median OS of 17.9 months, the irAEs were controllable [184]. The TRIPLET phase II clinical trial conducted by Zhang et al. [185] further explored the effectiveness of HAIC combined with camrelizumab and apatinib in aHCC with Barcelona Clinic Liver Cancer (BCLC) staging as stage C [185]. The confirmed ORR of this trial was 77.1% (RECIST v1.1 criteria), with a median PFS of 10.38 months [185]. Preliminary data suggested the potential of HAIC combined with targeted immunotherapy in the treatment of aHCC.

#### 3.5.4 Dual ICIs

The field of dual ICIs is gaining progress in research. The simultaneous application of PD‐1 and CTLA‐4 inhibitors can lead to synergistic effects. In clinical practice, this approach has already achieved notable progress in HCC [186].

The pivotal phase I/II trial CheckMate 040 evaluated the safety and efficacy of the combination of nivolumab and ipilimumab for the second‐line treatment of aHCC. This trial established three distinct treatment arms with different dosages and sequences of administration. Subsequent follow‐up revealed that arm A (nivolumab 1 mg/kg plus ipilimumab 3 mg/kg) exhibited an ORR of 34%, and the longest median PFS and OS among the arms [187]. Based on these findings, the combination of nivolumab and ipilimumab has been recommended for second‐line therapy for HCC in the ASCO guidelines [186]. Furthermore, at the 2024 ASCO, CheckMate 9DW showed that the combination of nivolumab and ipilimumab as a first‐line treatment for aHCC achieved an ORR of 36% and a median OS of 23.7 months [188]. This combination significantly enhanced patients' OS and ORR, with a controllable safety profile, suggesting its potential as first‐line treatment for aHCC.

The phase III trial HIMALAYA further explored the use of tremelimumab combined with durvalumab (STRIDE cohort) for first‐line management of HCC. The STRIDE cohort achieved a median OS of 16.4 months, compared with 13.8 months in the sorafenib group, and the ORR was more than triple than that of the sorafenib group [189]. The outcomes of HIMALAYA are promising and confirmed the significant role of immunotherapy in management of HCC.

The DUBHE‐H‐308 study, a phase II/III clinical trial by Qin et al. [190], assessed the efficacy and safety of QL1706—a dual PD‐1 and CTLA‐4 combination antibody, when combined with targeted therapy and/or chemotherapy as a first‐line treatment for aHCC. Preliminary results of DUBHE‐H‐308 are presented at the ESMO Congress, demonstrating an ORR of 35–36% with QL1706 when combined with targeted therapy and/or chemotherapy [190]. ZG005, a bispecific antibody targeting TIGIT/PD‐1, is the target of a phase II clinical trial (NCT06558227) in China, investigating its potential when combined with bevacizumab for first‐line treatment of aHCC. Several other dual ICI combinations are under clinical evaluation, and the advancements in these studies offer an expanded range of treatment options and hope for patients with aHCC (as illustrated in Table 5).

#### 3.5.5 Innovative Combination Therapies

The investigation of ICIs for HCC extends beyond existing therapies, with a multitude of clinical trials exploring innovative combination immunotherapies (as detailed in Table 5). Studies like NCT04251117, which integrates ICIs with a DNA vaccine and plasmid‐encoded IL‐12. NCT05155189, aligning ICIs with CAR‐T cell therapy. NCT06679985, examining the combination of ICIs with anti‐IL‐27 and targeted therapy, and NCT06695026, assessing the interaction between ICIs plus gene therapy and antiviral treatments. These clinical trials offer renewed optimism for HCC patients and highlight the evolving directions of immunotherapy advancements.

Preclinical studies are progressing as well. Histone deacetylases (HDACs), key enzymes in epigenetics that remove acetyl groups from histones, are increasingly being studied. Research by Tu et al. [191] indicated an elevated expression of HDAC1/2/3 in ICIs‐resistant mouse models, corresponding to the poor response to ICIs therapy in HCC patients with elevated HDAC1/2/3 expression. The combination of CXD101 (a selective HDAC1/2/3 inhibitor) with ICIs, significantly inhibited tumor growth without detectable side effects such as weight loss or organ abnormalities in mice [191]. The synergistic effect between novel targeted agents and ICIs is also under investigation. Cai et al. [192] observed a marked elevation in the number of tumor‐infiltrating cytotoxic CD8+ T cells in mice with knockdown of serine/arginine‐rich splicing factors following ICIs treatment, suggesting a potential boost in antitumor effects. Zhou et al. [193] reported that by blocking4‐Acetylaminobutyric acid in CD8+ T cells, a molecule of the AKT1 signaling pathway, may be a novel immunotherapeutic strategy when combined with ICIs therapy. Furthermore, Cao et al. [194] demonstrated in HCC mouse models that PARP1 inhibitors (olaparib) could enhance the effectiveness of PD‐1 inhibitor. The role of chemokine receptors in cellular signaling is also under investigation, with preclinical studies showing that targeting these receptors (CXCR4, CX3CR1) in combination with ICIs can amplify antitumor effects [195, 196]. These investigations provide insights into advanced liver cancer treatments and pave the way for precision therapy in the future.

### 3.6 Bladder Cancer

Bladder cancer is the commonest urological malignancy after prostate cancer, with the 9th highest morbidity and 13th highest mortality globally, more than 90% of bladder cancer are urothelial carcinoma (UC) [1]. Based on the depth of tumor infiltration, bladder cancer is classified as muscle invasive bladder cancer (MIBC) and nonmuscle invasive bladder cancer (NMIBC), of which NMIBC accounts for about 70%, yet 25–30% may progress to MIBC. Currently, the standard treatment for NMIBC is transurethral resection of bladder tumor (TURBT) in combination with intravesical instillation of chemotherapy or Bacillus Calmette‐Guérin (BCG), while the standard treatment for MIBC is radical cystectomy combined with pelvic lymph node dissection [197, 198]. Comprehensive systemic therapies, including chemotherapy, radiation therapy and so on, are used to treat advanced bladder cancer. However, chemotherapy is ineffective and nearly half of the patients are intolerant to cisplatin‐based chemotherapy, while RT is accompanied by numerous complications, such as radiation proctitis, radiation cystitis, and so on [199]. With the advent of the immunotherapy era, the efficacy of bladder cancer treatment has been significantly improved, ICIs can cover treatment for all stages of bladder cancers. The completed and ongoing clinical studies of ICIs in bladder cancer are displayed in Table 6.

**TABLE 6:** Clinical trials of ICIs for bladder cancer.

Tumor type	Clinical trial name	Registration number	Country	Completion date	Starting date	LOT	Phase	Combination type	Regimens	No. of patients	mPFS (months)	mOS (months)	ORR	Grade 3/4 TRAEs
**Clinical trials with results of ICIs for bladder cancer**														
MIBC	PURE‐01	NCT02736266	Italy	2022/9/23	2017/2/27	NA	II	Single‐agent	Pembrolizumab	50	NA	NA	42.0%	NA
Advanced UC	AVELIN Bladder 100	NCT02603432	USA	2023/3/28	2016/4/25	First‐line	III	Single‐agent	Avelumab	700	3.7	21.4	NA	47.4%
Advanced UC	IMvigor 210	NCT02108652	USA	2023/2/28	2014/5/31	First‐line	II	Single‐agent	Atezolizumab	123	2.7	15·9	23.0%	16.0%
Advanced UC	KEYNOTE‐361	NCT02853305	Australia	2022/9/15	2016/9/15	First‐line	III	Single‐agent	Pembrolizumab	1010	8.3	17·0	NA	NA
Advanced UC	KEYNOTE‐045	NCT02256436	USA	2020/10/1	2014/10/22	Second‐line	III	Single‐agent	Pembrolizumab	542	NA	10.3	NA	15.0%
Advanced UC	CheckMate 275	NCT02387996	USA	2021/11/12	2015/3/9	Second‐line	II	Single‐agent	Nivolumab	270	NA	7.0	19·6%	18.0%
Advanced UC	—	NCT01772004	USA	2019/12/16	2013/1/31	Second‐line	Ib	Single‐agent	Avelumab	54	2.7	13.7	18.2%	6.8%
MIBC	BLASST‐1	NCT03294304	USA	2021/7/1	2018/1/29	NA	II	Chemotherapy + ICIs (anti‐PD‐1)	Gemcitabine‐cisplatin + nivolumab	41	NA	NA	65.8% (pCR)	20.0%
MIBC	NABUCCO	NCT03387761	Netherlands	2021/9/13	2018/1/15	NA	I	Dual‐ICIs (anti‐PD‐1 + anti‐CTLA‐4)	Nivolumab + ipilimumab	24	NA	NA	cCR: 46.0%	55.0%
MIBC	ANZUP 1502	NCT02662062	USA	2024/1/1	2016/8/1	NA	II	CRT + ICIs(anti‐PD‐1)	CRT + pembrolizumab	28	NA	39.0	88.0% (CR)	21.4%
MIBC	PrE0807	NCT03532451	USA	2019/3/22	2022/10/5	NA	Ib	Targeted therapy (anti‐KIR) + ICIs (anti‐PD‐1)	Lirilumab + nivolumab vs. nivolumab	43	NA	2‐year OS: 89.0 vs. 82.0%	pCR: 21.0 vs. 17.0%	7.0 vs. 0.0%
**Ongoing clinical trials of ICIs for bladder cancer**														
NMIBC	KEYNOTE‐057	NCT02625961	USA	Ongoing	2016/2/10	NA	Phase II	Single‐agent	Pembrolizumab	320	NA	NA	NA	NA
BCG‐Unresponsive NMIBC	—	NCT02844816	USA	Ongoing	2017/3/13	NA	Phase II	Single‐agent	Atezolizumab	172	NA	NA	NA	NA
MIBC	—	NCT03520491	USA	Ongoing	2018/4/25	NA	Phase II	Single‐agent	Nivolumab	45	NA	NA	NA	NA
MIBC	IMvigor011	NCT04660344	Brazil	Ongoing	2021/5/3	NA	Phase III	Single‐agent	Atezolizumab	800	NA	NA	NA	NA
MIBC	—	NCT05406713	USA	Ongoing	2022/7/13	NA	Phase II	Single‐agent	Pembrolizumab	46	NA	NA	NA	NA
HR NMIBC	​KEYNOTE‐676	NCT03711032	USA	Ongoing	2018/12/24	NA	Phase III	BCG + ICIs(anti‐PD‐1)	BCG + pembrolizumab	1397	NA	NA	NA	NA
HR NMIBC	ALBAN	NCT03799835	Belgium	Ongoing	2019/1/17	NA	Phase III	BCG + ICIs (anti‐PD‐L1)	BCG + atezolizumab	516	NA	NA	NA	NA
MIBC	—	NCT02989584	—	Ongoing	2016/12/20	NA	Phase I/II	Chemotherapy + ICIs (anti‐PD‐L1)	Gemcitabine‐cisplatin + atezolizumab	54	NA	NA	NA	NA
MIBC	—	NCT03661320	USA	Ongoing	2018/11/6	NA	Phase III	Chemotherapy + ICIs (anti‐PD‐1)	Gemcitabine‐cisplatin+nivolumab	861	NA	NA	NA	NA
MIBC	KEYNOTE‐866	NCT03924856	USA	Ongoing	2019/6/13	NA	Phase III	Chemotherapy + ICIs (anti‐PD‐1)	Gemcitabine‐cisplatin + pembrolizumab	907	NA	NA	NA	NA
MIBC	NEXT	NCT03171025	USA	Ongoing	2017/7/10	NA	Phase II	Chemoradiotherapy + ICIs (anti‐PD‐1)	Chemoradiotherapy + nivolumab	200	NA	NA	NA	NA
MIBC	—	NCT03775265	USA	Ongoing	2019/6/3	NA	Phase III	Chemoradiotherapy + ICIs (anti‐PD‐L1)	Chemoradiotherapy + atezolizumab	475	NA	NA	NA	NA
MIBC	—	NCT04241185	USA	Ongoing	2020/5/19	potential first‐line	Phase III	Chemoradiotherapy + ICIs (anti‐PD‐1)	Chemoradiotherapy + pembrolizumab	520	NA	NA	NA	NA
MIBC	—	NCT05072600	China	Ongoing	2021/12/7	potential first‐line	Phase II	Chemoradiotherapy + ICIs (anti‐PD‐1)	Chemoradiotherapy + pembrolizumab	54	NA	NA	NA	NA
NMIBC	—	NCT05203913	Italy	Ongoing	2023/5/1	NA	Phase II	Chemoradiotherapy + ICIs (anti‐PD‐1)	Chemoradiotherapy + nivolumab	32	NA	NA	NA	NA
MIBC	KEYNOTE‐905/​EV‐303	NCT03924895	USA	Ongoing	2019/7/24	NA	Phase III	ADC + ICIs (anti‐PD‐1)	Enfortumab vedotin + pembrolizumab	595	NA	NA	NA	NA
MIBC	INTerpath‐005	NCT06305767	USA	Ongoing	2024/3/28	NA	Phase I/II	ADC + mRNA + ICIs (anti‐PD‐1)	Enfortumab vedotin + mRNA‐4157 + pembrolizumab	230	NA	NA	NA	NA
MIBC	—	NCT06470282	USA	Ongoing	2024/11/30	NA	Phase Ib/II	ADC + RT + ICIs (anti‐PD‐1)	Enfortumab vedotin + radiotherapy + pembrolizumab	47	NA	NA	NA	NA
MIBC	—	NCT04289779	USA	Ongoing	2020/5/21	NA	Phase II	Targeted therapy (TKI) + ICI (anti‐PD‐L1)	Cabozantinib + atezolizumab	46	NA	NA	NA	NA
MIBC	—	NCT06059547	Italy	Ongoing	2023/9/6	NA	Phase II	Targeted therapy (anti‐GDF‐15) + ICI (anti‐PD‐1)	Visugromab + nivolumab	30	NA	NA	NA	NA
BCG‐Unresponsive NMIBC	—	NCT05843448	USA	Ongoing	2023/4/19	NA	Phase I	Vaccine + ICI (anti‐PD‐1)	IO102‐IO103 + pembrolizumab	30	NA	NA	NA	NA
BCG‐Unresponsive NMIBC	—	NCT04164082	USA	Ongoing	2020/3/18	NA	Phase II	Antimetabolite drug (pyrimidine nucleoside analog) + ICI (anti‐PD‐1)	Gemcitabine + pembrolizumab	161	NA	NA	NA	NA

The trials in the table are arranged in the order of their appearances in the main text. For trials not explicitly discussed in the text, they are ordered chronologically by trial initiation dates (ascending order).

*Data sources*: ClinicalTrials.gov (clinicaltrials.gov), PubMed, Wanfang Data (https://www.wanfangdata.com.cn/), ESMO (European Society for Medical Oncology), and ASCO (American Society of Clinical Oncology—ASCO).

Abbreviations: anti‐GDF‐15, antigrowth differentiation factor 15.; anti‐KIR, anti‐killer‐cell immunoglobulin‐like receptor; BCG‐unresponsive NMIBC, Bacillus Calmette‐Guérin‐unresponsive nonmuscle‐invasive bladder cancer; HR NMIBC, high‐risk nonmuscle‐invasive bladder cancer; MIBC, muscle‐invasive bladder cancer; UC, urothelial carcinoma.

#### 3.6.1 ICIs Monotherapy

Adjuvant intravesical immunotherapy after TURBT has been recommended to intermediate and high‐risk NMIBC and carcinoma in situ (CIS) to induce local immune responses in order to prevent recurrence and control progression. BCG is the primary medication used in adjuvant perfusion immunotherapy, and several studies have confirmed that for high‐risk patients, intravesical BCG perfusion reduces the risk of progression by 27% compared with chemotherapeutic agents [200]. Notably, pembrolizumab is indicated for the management of patients with BCG‐resistance, high‐risk NMIBC with CIS who are ineligible for or have decided not to undergo cystectomy according to 2024 NCCN guidelines. In a prospective phase II study (KEYNOTE‐057), pembrolizumab was applied to BCG‐resistance, CIS NMIBC patients, the results exhibited CR at 3 months in 41% of patients; 13% of patients developed grade 3 or higher irAEs [201].

Neoadjuvant therapy and adjuvant therapy in MIBC patients is significantly common. The PURE‐01 study, a neoadjuvant therapy for MIBC with pembrolizumab, demonstrated a pCR rate of 42%, and a stage‐decrease rate (<pT2) of 54% [202]. Moreover, 95 patients received atezolizumab as neoadjuvant therapy in the ABACUS trial indicated that 31% of patients achieved pCR, with grade 3 or 4 irAEs experienced in 11% of patients [203]. However, further evidence‐based study is required for neoadjuvant immunotherapy. MIBC patients with high‐risk recurrence, including pT3‐T4 staging, lymph node positivity or intolerance to cisplatin are recommended for adjuvant therapy. CheckMate274 is a phase III, multicenter RCT, which reported nivolumab versus placebo for muscle‐invasive UC in patients with high‐risk recurrence. DFS was significantly better in the nivolumab group than in the control group (22.0 vs. 10.9 month; HR: 0.71) and grade ≥3 irAEs occurred in 18.2 and 7.2% in the nivolumab and placebo group, respectively [204]. Furthermore, another phase III RCT showed that adjuvant treatment of MIBC with atezolizumab and control resulted in a prolonged DFS for atezolizumab versus control, 19.4 versus. 16.6 months, respectively (HR 0.89; *p* = 0.24) [205]. Currently, nivolumab is the preferred adjuvant immunotherapy agent in the MIBC population with high‐risk recurrence. The IMvigor 011 study (NCT04660344) focusing on adjuvant immunotherapy for ctDNA‐positive patients after radical cystectomy is currently underway.

ICIs monotherapy is primarily used as maintenance therapy following platinum‐based chemotherapy. Powles et al. [206] conducted a phase III study (NCT02603432) of avelumab for maintenance therapy in patients with locally progressive or metastatic UC after first‐line platinum‐based chemotherapy, and the results demonstrated that median OS of avelumab and control were 21.4 and 14.3 months, respectively (HR: 0.60; *p* = 0.001). For platinum‐intolerant UC of the bladder, pembrolizumab and atezolizumab (only for patients whose tumors express PD‐L1) may be considered as first‐line treatment. The IMvigor 210 study demonstrated that atezolizumab for patients with cisplatin‐intolerant advanced MIBC had an ORR of 23% and a median OS of 15.9 months [207]. In addition, a phase III study (KEYNOTE‐361) applied pembrolizumab as first‐line treatment for patients with previously untreated advanced UC, a 15.6 months (95% CI 12.1–17.9) median OS was demonstrated with pembrolizumab monotherapy, with diarrhea, fatigue and hyponatremia as the commonest grade 3 or higher irAEs [208]. Currently, US FDA has approved pembrolizumab, nivolumab, and avelumab for patients with advanced UC who have unsatisfactory platinum‐based chemotherapy (www.fda.gov). Joaquim Bellmunt et al. [209] reported pembrolizumab as second‐line treatment for advanced UC in the KEYNOTE‐045 study (NCT02256436), the median OS in the total population was 10.3 months with pembrolizumab, as compared with 7.4 months with chemotherapy (HR: 0.73; *p* = 0.002). Nivolumab has likewise been used as a second‐line treatment in advanced UC, with a multicenter, single‐arm phase II clinical study (CheckMate 275, NCT02387996) revealing an ORR of 19.6% in 270 patients treated with nivolumab [210]. Avelumab was used in the treatment of metastatic UC in a phase Ib, multicenter, expansion cohort study (NCT01772004), with a median PFS of 11.6 weeks and a median OS of 13.7 months in patients with PD‐L1‐positive tumors [211].

#### 3.6.2 Synergistic Treatment Approach

Combined immunotherapy regimens are increasingly applied in the perioperative treatment of MIBC and advanced UC, and there is an apparent trend toward sequential, combination treatment modalities, not only because immunotherapy has been shown to be synergistic with other regimens, such as chemotherapy and RT, but it also markedly improves the CR rate of patients [212, 213].

A total of 41 patients were enrolled in BLASST‐1 (NCT03294304) with nivolumab and gemcitabine‐cisplatin (GC) neoadjuvant therapy, and 27 (65.8%) MIBC patients achieved pCR and the overall rates of grade ≥3 irAEs was 20% [214]. The BGB‐A317‐2002 study published in 2022 indicated that MIBC patients receiving tislelizumab combined with GC as neoadjuvant therapy have a pCR rate of 58.8%, with no grade ≥3 irAEs seen [215]. Dual‐ICIs regimens are also seen in neoadjuvant therapy for MIBC patients, such as the NABUCCO study. After receiving ipilimumab and nivolumab, 11 (46%) patients reached pCR and 55% of patients experienced grade 3–4 irAEs [216]. It is worth noting that there is currently limited evidence for adjuvant immunotherapy. In addition to the application of nivolumab monotherapy for MIBC mentioned above, more ICIs treatment regimens need to be developed, especially combined immunotherapy.

In cisplatin‐tolerant populations, cisplatin‐based chemotherapy combined with nivolumab is currently the first‐line treatment for metastatic UC. According to the results of Checkmate 901 (NCT03036098), there was a benefit in PFS and OS in combination with Nivolumab and GC regimen, with a median OS for 21.7 months (95% CI, 18.6–26.4), and a PFS rate of 34.2% at 12 months, and grade 3 or above irAEs occurred in 61.8% of patients [217]. While for cisplatin‐intolerant patients, carboplatin can be considered as an alternative, such as the gemcitabine–carboplatin and methotrexate–carboplatin–vinblastine regimen [218].

#### 3.6.3 Novel Combination Treatment

Notably, ADCs have gained prominence in recent years as a class of therapeutic agents to selectively deliver potent cytotoxic drugs to tumor cells expressing specific antigens through monoclonal antibodies. Enfortumab vedotin is one of the ADCs, achieved an ORR of 73.3% and median OS of 26.1 months in combination with pembrolizumab as a first‐line treatment for patients with locally advanced and metastatic UC who have unsatisfactory platinum‐based chemotherapy [219]. Furthermore, disitamab vedotin (DV) is a novel humanized anti‐HER2 ADCs agent, which was studied in RC48‐C014 study. Patients were systemically treated with RC48‐ADC and toripalimab, resulted in an ORR of 75%, the ORR was 100% for patients with HER‐2 (3+), 77.8% for HER‐2 (2+), 66.7% for HER‐2 (1+), and 50% for HER‐2 (0), respectively [220].

ICIs have brought new options to the treatment for bladder cancer, ICIs monotherapies are another treatment option for NMIBC patients with BCG‐resistance or recurrence. In the perioperative treatment of MIBC, neoadjuvant immunotherapy significantly increased the pCR rate and has an excellent safety profile. The efficacy of adjuvant immunotherapy with nivolumab has been affirmed and recommended by guidelines. Combined immunotherapy regimens are still being explored for perfusion therapy in NMIBC, and better therapeutic modalities are expected to fill the data gaps. Perioperative sequential and combination therapy is the future trend of MIBC treatment, and a variety of combination regimens, such as dual‐ICIs, immune‐RT, immunochemotherapy, and immuno‐targeted therapy are underway. Furthermore, the continuous improvement of treatment efficacy also provides more opportunities for bladder‐preserving treatment. Immunochemotherapy is currently the first‐line treatment option for advanced UC, but with today's emergence of various new agents, such as FGFR inhibitors, CDK4/6 inhibitors, as well as ADCs, treatment option for advanced UC is full of promise and is a hot topic for future research.

## 4 Safety Profiles and Management of irAEs

ICIs represent a double‐edged sword in cancer treatment. While these agents have revolutionized the treatment landscape across diverse malignancies by reactivating antitumor immunity, their therapeutic benefits are counterbalanced by the risk of immune‐mediated inflammatory reactions in virtually any organ system, collectively termed irAEs [221, 222]. The Common Terminology Criteria for Adverse Events categorizes irAEs into four grades, with grade 3 or 4 events necessitating ICIs discontinuation and often warranting permanent cessation due to life‐threatening complications [223]. Consequently, a comprehensive understanding of irAEs associated with distinct ICIs, coupled with evidence‐based management strategies, is imperative for clinicians to optimize therapeutic outcomes while minimizing harm.

### 4.1 Drug‐Specific Side Effects of US FDA‐Approved ICIs

#### 4.1.1 CTLA‐4 Inhibitor

Ipilimumab, the first ICI approved by the US FDA, is currently indicated as monotherapy for unresectable or metastatic melanoma [224]. Clinical trials have demonstrated that ipilimumab monotherapy improves OS in patients with metastatic melanoma, establishing its significance in melanoma treatment [225]. However, its safety profile warrants particular attention. A meta‐analysis of 22 clinical trials by Bertrand et al. [226] revealed that CTLA‐4 inhibitor‐related adverse events predominantly involve dermatologic and gastrointestinal toxicities, with endocrine and hepatic toxicities being less frequent. Gastrointestinal irAEs are a critical concern for CTLA‐4 inhibitors, with diarrhea being the most commonly reported symptom [226]. Specifically, ipilimumab monotherapy is associated with diarrhea in approximately 33.1% of patients, of whom 11.6% develop colitis [227]. Dermatologic toxicities are also prevalent, manifesting as pruritus (35.4%) and rash (32.8%) [227].

#### 4.1.2 PD‐1 Inhibitors

Nivolumab and pembrolizumab have been approved by the US FDA for the treatment of multiple solid tumors and hematologic malignancies. Systematic reviews and meta‐analyses indicate that PD‐1 inhibitors significantly increase the risk of hypothyroidism, pneumonitis, colitis, and hypophysitis, though severe organ‐specific irAEs remain uncommon overall [228]. A pooled analysis of Nivolumab demonstrates that the most frequent all‐grade irAEs involve the skin, gastrointestinal tract, and endocrine system, while grade ≥3 irAEs predominantly involve the liver, gastrointestinal tract, and skin [229]. Toxicity profiles vary markedly across tumor types. Rahma et al. [230] reported a 20.1% incidence of grade 3–5 irAEs with nivolumab, with lower rates observed in NSCLC compared with renal cell carcinoma or melanoma. Notably, a meta‐analysis by Liang et al. [231] showed that nivolumab carries a relatively lower risk of grade 3–5 irAEs in NSCLC patients. Pembrolizumab exhibits a lower overall incidence of grade 3–5 irAEs, particularly reduced in melanoma compared with NSCLC [230], underscoring the necessity for tumor type‐specific irAEs monitoring.

Cemiplimab, approved by the US FDA between 2018 and 2021 for cutaneous squamous cell carcinoma, basal cell carcinoma and NSCLC with high PD‐L1 expression [232, 233], demonstrates distinct toxicity patterns. In the NCT03132636 trial for locally advanced basal cell carcinoma, the most common irAEs included hypothyroidism and immune‐mediated colitis, while severe irAEs primarily involved colitis and adrenal insufficiency [234]. NSCLC patients treated with cemiplimab exhibit the lowest risk of all‐grade irAEs among PD‐1/PD‐L1 inhibitors [231].

Dostarlimab, initially approved in 2021 for dMMR recurrent or advanced solid tumors, received expanded approval in 2023 for dMMR endometrial cancer [235]. In the GARNET trial, the most frequent irAEs in dMMR solid tumor patients were hypothyroidism, elevated alanine aminotransferase (ALT), and arthralgia [236]. Subsequent analyses of the endometrial cancer cohort within the GARNET trial revealed similar irAEs profiles [235].

#### 4.1.3 PD‐L1 Inhibitors

Atezolizumab has been approved for the treatment of NSCLC, small cell lung cancer (SCLC), TNBC, HCC, UC, and others [237, 238, 239, 240]. The most common organ‐specific irAEs following atezolizumab treatment are gastrointestinal irAEs, respiratory/thoracic irAEs, and hematologic/lymphatic system irAEs [241]. Analysis of its safety profile shows that age significantly affects the toxicity spectrum. In patients aged 65–85 years, the most common irAEs are urinary tract, cardiac, and metabolic irAEs. In contrast, patients aged 18–64 mainly experience immune system, ear/labyrinth and reproductive system/breast irAEs [241]. It is also noteworthy that the type of tumor can influence the safety profile. In patients with NSCLC, atezolizumab monotherapy increases the risk of pneumonia, adrenal insufficiency, and thyroid dysfunction compared with previous data [237]. In SCLC patients receiving combination chemotherapy, the incidence of rash, hypothyroidism, and hepatitis is increased [242]. In patients with TNBC, combination therapy of atezolizumab and chemotherapy compared with chemotherapy alone increases the risk of diarrhea, hypothyroidism, and hyperthyroidism [243].

Durvalumab has been approved by the US FDA for unresectable stage III NSCLC, biliary tract cancer (BTC) and HCC [244, 245, 246, 247]. Its safety profile shows tumor type dependency. Based on the phase III PACIFIC trial, the most common irAEs in patients with unresectable stage III NSCLC treated with durvalumab were cough, fatigue, pneumonia/radiation pneumonitis, upper respiratory tract infection, dyspnea, and rash [246]. In patients with HCC and BTC, the frequently reported irAEs were fatigue, nausea, constipation, anorexia, abdominal pain, rash, and fever [245, 247].

#### 4.1.4 LAG‐3 Inhibitors

Based on the results of RELATIVITY‐047, the immunotherapy regimen of relatlimab (a LAG‐3 inhibitor) in combination with Nivolumab was approved by the US FDA in March 2022 for the treatment of unresectable or metastatic melanoma [248]. This trial indicated that grade 3–4 irAEs in the combination therapy were primarily elevated lipase, ALT, and aspartate aminotransferase (AST) and fatigue [248]. A multicenter retrospective study on advanced melanoma revealed that irAEs associated with the nivolumab/relatlimab combination were predominantly endocrine and gastrointestinal irAEs [249]. In the recently published phase II NICHE‐3 study, patients with locally advanced dMMR colorectal cancer receiving neoadjuvant nivolumab/relatlimab therapy mainly experienced infusion‐related reactions, thyroid dysfunction, and fatigue. These differences may stem from variations in treatment modalities and the TME. Future research should expand sample sizes and conduct cross‐tumor comparative studies to elucidate the toxicity of LAG‐3 inhibitors [250].

#### 4.1.5 Combination Therapies

Due to the limited efficacy of monotherapy in antitumor treatment, an increasing number of combination therapies have been explored and implemented. Among them, the combination of nivolumab and ipilimumab has been approved by the US FDA for melanoma, malignant pleural mesothelioma, and HCC [10, 251]. However, the synergistic effect of dual ICIs significantly increases the risk of severe irAEs [252]. A meta‐analysis has shown a significantly higher risk of severe irAEs with the combination of nivolumab and ipilimumab compared with PD‐1 monotherapy and other combination regimens [253]. In clinical practice, fatal irAEs cases have been observed, such as severe myositis following ipilimumab/nivolumab treatment, which manifests similar to that of bulbar palsy, requiring mechanical ventilation support [254]. Studies have shown that combination therapy can exacerbate organ‐specific toxicities. Humanized mouse models have shown that dual immunotherapy of PD‐1/CTLA‐4 inhibitors induces severe interstitial nephritis and vasculitis compared with monotherapy, with CD4+ T cell aggregation in the affected tissues, consistent with the renal pathological phenotype in patients [255]. These findings highlight the necessity of risk stratification and toxicity monitoring in combination therapy.

### 4.2 Safety Management Strategies for ICIs

#### 4.2.1 Organ‐Specific Monitoring

**FIGURE 2:**
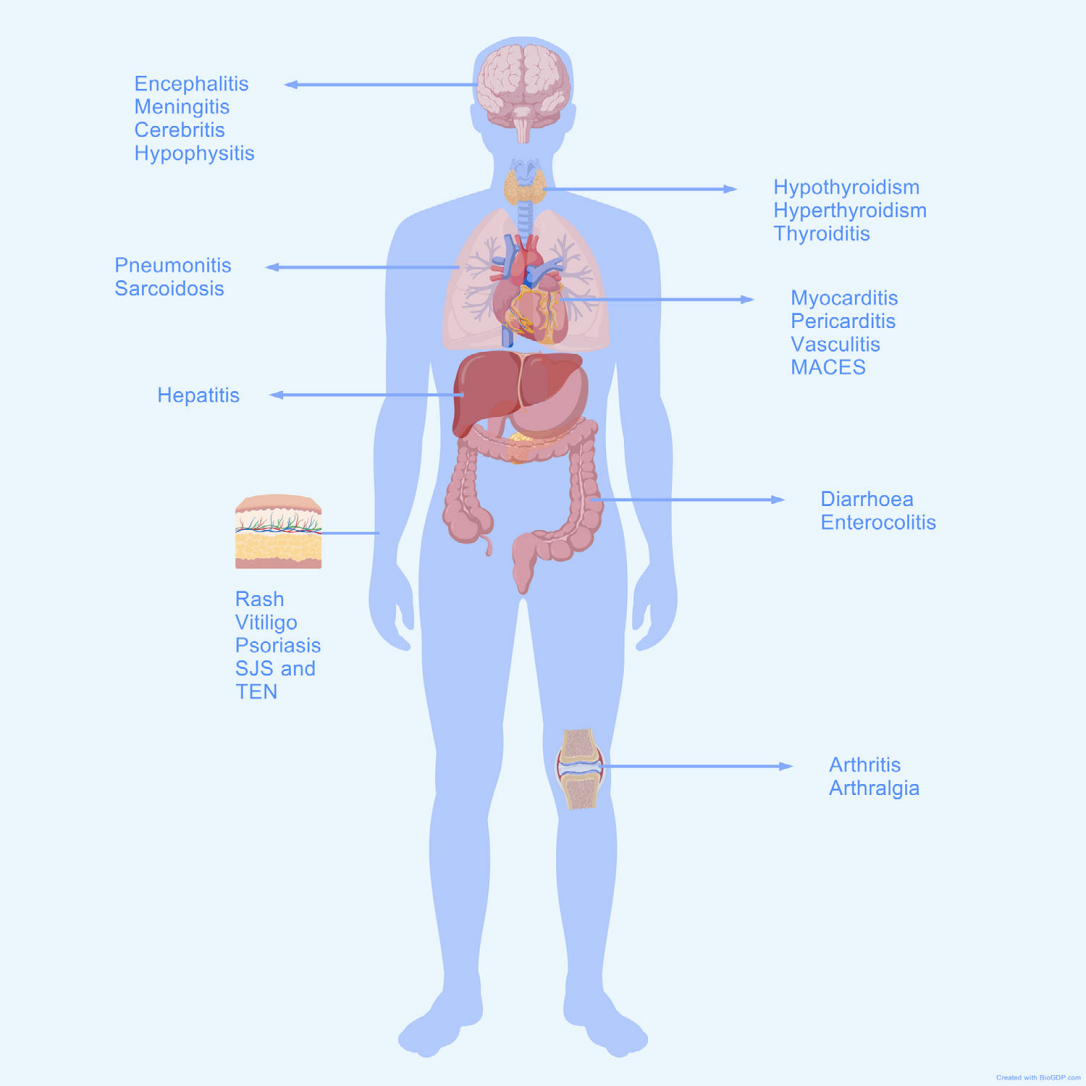
The organ‐specific toxicity atlas of immune‐related adverse events. (The figure is redrawn from Figure 2 in Chen et al.’s article [224], originally published under a CC BY 4.0 license.) This figure systematically categorizes organ‐specific immune‐related adverse events (irAEs) associated with immune checkpoint inhibitor therapy. The atlas classifies irAEs by affected organ systems, including central nervous system (encephalitis, meningitis, cerebritis, hypophysitis), respiratory system (pneumonitis, sarcoidosis), liver (hepatitis), skin (rash, vitiligo, psoriasis, SJS/TEN), endocrine system (hyperthyroidism, thyroiditis), cardiovascular system (myocarditis, pericarditis, vasculitis, MACES), gastrointestinal system (diarrhea, enterocolitis), and musculoskeletal system (arthritis, arthralgia). Abbreviations: ATG, antithymocyte globulin; MACEs, major adverse cardiac events; SJS, Stevens–Johnson syndrome; TEN, toxic epidermal necrolysis.

Organ‐specific monitoring is crucial for early detection and management of irAEs. Various ICIs can cause irAEs in multiple organ systems, among which the skin, gastrointestinal tract and endocrine system (such as shown in Figure 2) are the most commonly affected [224]. The tissue and organ toxicities of ICIs are gender‐specific, with male patients having higher risks of respiratory and urological toxicities, while female patients are more susceptible to reproductive toxicities [241]. When using ICIs, regular monitoring of organ functions, including liver, kidney, heart, and thyroid functions, should be conducted [256]. For patients receiving combination therapies, which are associated with a higher risk of severe irAEs, more frequent monitoring is required. Imaging examinations such as CT scans, MRI, and endoscopy can help identify organ‐specific toxicities, especially in asymptomatic patients [256]. In addition, closely monitoring symptoms related to specific organs is essential. For example, patients should be educated to promptly report any signs of skin toxicity, such as rashes or pruritis, as well as gastrointestinal symptoms, such as diarrhea or abdominal pain.

#### 4.2.2 Risk Factors and Biomarkers for Severe irAEs

The identification of risk factors and biomarkers for severe irAEs is critical for optimizing ICIs therapy. Patient‐specific factors such as advanced age, female sex, preexisting autoimmune diseases (AIDs), and prior antibiotic use are strongly associated with increased irAEs risk, particularly in patients receiving ICIs combination therapy [257, 258]. Notably, elderly patients with preexisting AIDs face a higher risk of severe irAEs [258]. However, elderly patients may exhibit lower irAEs incidence, potentially due to age‐related immune senescence, conservative management, or underreporting [259]. Treatment‐related factors, including ICIs combination regimens, further amplify irAEs risks [257, 258].

Blood‐based biomarkers provide valuable insights. Elevated baseline absolute lymphocyte count and eosinophil count, alongside lower NLR and platelet‐to‐lymphocyte ratio, are linked to higher irAEs risk, though optimal thresholds remain undefined [260]. Monocyte‐related markers further refine predictions. A high monocyte‐to‐lymphocyte ratio predicts shorter PFS (HR = 1.5) and OS (HR = 1.52) [261]. Functional immune profiling reveals that severe irAEs are associated with monocyte dysfunction, including reduced IL‐10 production poststimulation, which impairs T‐cell regulation and drives T‐cell expansion [262]. Conversely, higher baseline T‐cell diversity predicts superior treatment response and lower irAEs risk, suggesting a protective role of immune repertoire breadth [263].

Organ‐specific biomarkers enhance early detection. In nephritis, a urine proteomic signature (IL‐5 + Fas) achieves an AUC of 0.94 for diagnosing ICIs‐associated acute interstitial nephritis, enabling noninvasive monitoring [264]. For pneumonitis, serum Krebs von den Lungen‐6 (KL‐6) serves as a robust screening biomarker in NSCLC patients, though its utility in NSCLC‐specific pneumonitis is limited [265]. As for myocarditis, the expansion of CXCL9/10+CCR2+ macrophages and CXCR3hi CD8+ T lymphocytes is linked to the pathogenesis of ICI‐associated myocarditis, highlighting the therapeutic potential of CXCR3 pathway modulation [266].

Gut microbiome dynamics are emerging as predictive biomarkers. Dysbiosis characterized by increased pathobionts and decreased Ruminococcaceae abundance predisposes patients to severe irAEs, particularly colitis [267]. Longitudinal microbiome analysis reveals acute perturbations during irAEs development, emphasizing its role in monitoring irAEs [268].

The above findings demonstrated the multifactorial nature of irAEs. Integrating demographic, hematologic, microbial, and molecular factors, prospective validation of these biomarkers is essential to guide personalized risk stratification management.

#### 4.2.3 Novel Strategies for the Management of irAEs

Adiponectin can reduce proinflammatory T cells in the colon, thereby alleviating ICIs‐induced colitis without affecting antitumor immunity. Clinical trials have shown that adiponectin achieves a 100% CR rate and reduces glucocorticoid dependence [269]. In a mouse model of liver cancer, glucocorticoid pretreatment did not weaken the antitumor efficacy of dual ICIs therapy, supporting its feasibility in managing severe irAEs [270]. Antiviral therapy significantly reduces the incidence of severe irAEs in HBV‐positive HCC patients receiving ICIs, highlighting the importance of viral control in managing irAEs [271]. Research on the molecular mechanisms of irAEs has revealed key intervention targets, such as cytokines (IL‐6, TNF‐α), integrins, and microbiome regulation, suggesting the efficacy of targeted antibodies or modulators to reduce toxicity while preserving antitumor activity [272]. Novel tumor‐responsive nanoparticles selectively release active components in the TME, reducing systemic immune toxicity and extending survival [273]. In addition, glucagon‐like peptide‐1 (GLP‐1) receptor agonists can reduce the risk of major cardiovascular events in cancer patients treated with ICIs (with a median follow‐up of 12 months, the incidence of major adverse cardiovascular events was significantly lower than the control group) [274]. These novel strategies provide multidimensional solutions for balancing efficacy and safety.

## 5 Conclusion and Prospects

Spanning four decades, the introduction of ICIs has revolutionized cancer treatment. The development of PD‐1/PD‐L1 inhibitors and CTLA‐4 inhibitors has provided significant and long‐lasting clinical benefits for patients across a spectrum of cancers. Variations in response rates to ICIs across different tumor types, primary and metastatic tumors, and distinct cancer subtypes within the same tumor reflect the tumor heterogeneity and complexity of TME [275]. The synergistic use of ICIs with other immunotherapies, chemotherapies, or targeted therapies has also proven effective in a range of cancers. However, the irAEs associated with immunotherapy is a notable concern. Patients receiving CTLA‐4 inhibitors are at a higher risk for severe irAEs compared with those on PD‐1/PD‐L1 inhibitors, and the combination of CTLA‐4 inhibitors and PD‐1/PD‐L1 inhibitors can increase the risk of irAEs [276]. This emphasizes the necessity for predictive biomarkers to enhance patient selection, optimizing treatment efficacy while minimizing irAEs.

US FDA has approved PD‐L1, TMB, and dMMR/MSI‐H as the three predictive biomarkers for ICIs’ efficacy. The potential of tumor‐infiltrating lymphocytes as an additional biomarker for ICIs is gaining recognition. Yet, challenges remain in overcoming the limitations of these biomarkers and in setting standardized thresholds across tumor types. The clinical application of blood‐based biomarkers for ICIs, including ctDNA, NLR, and costimulatory molecules, is increasingly recognized [277]. With advancements in genetic sequencing and multiomics technologies, the predictive potential of genetic mutations is under investigation. Researchers, including Glitza et al. [278], have underscored the gut microbiome's role as a predictive marker for ICIs in a multicenter RCT, stressing the importance of combining microbial interventions with ICIs treatments. These biomarkers offer novel avenues for precision medicine, enabling a more accurate prediction of patient responses and outcomes. The development of multimodal biomarker strategies is expected, providing a more comprehensive reference for clinical decision‐making.

Furthermore, the findings of new immune checkpoint targets and the creation of innovative combination treatments may be significant strategies in the future. Inhibitors of emerging targets like TIGIT, TIM‐3, and VISTA have demonstrated preliminary antitumor effects in early clinical trials. Preliminary experiments have also indicated the mechanisms by which TIGIT and TIM‐3 inhibitors, in combination with PD‐1/PD‐L1 inhibitors, may enhance antitumor effects, with early clinical trials in progress [279]. Early clinical trials and preclinical studies are exploring innovative combination strategies. For example, growth differentiation factor 15 (GDF‐15), part of the TGFβ superfamily, has shown sustained antitumor effects when combined with PD‐1 inhibitors in the GDFATHER‐1/2a trial [280]. The combination of oncolytic virus talimogene laherparepvec and ipilimumab has also shown durable improved response rates in early clinical trials [281]. Autophagy, crucial for the degradation of obsolete cellular components, offers potential in overcoming therapeutic resistance to ICIs [282]. The combination of antigen vaccines and ICIs has shown promising efficacy in early clinical trials, and recent animal studies suggest that neoantigen vaccines combined with dual ICIs can elicit superior antitumor effects, although further clinical validation is required [283, 284]. CD47, a natural immune checkpoint, may play a key role in tumor immune evasion, and the combination of CD47 antibodies with rapamycin has been shown to enhance antitumor effects in mice [285]. Additionally, targeting the gut microbiome, ataxia‐telangiectasia mutated (ATM), and noncoding RNA in conjunction with ICIs may offer new therapeutic strategies, as indicated by basic and animal experiments [286, 287, 288].

In conclusion, while ICIs have marked a new era in cancer treatment, optimizing immunotherapy protocols, including drug dosage, timing, and combination strategies, is necessary to reduce side effects and improve outcomes. Concurrently, exploring new biomarkers to predict treatment responses and side effects will aid in the implementation of personalized immunotherapy.

## Author Contributions

Zhijun Chen: Conceptualization, writing—original draft and revising. Zihan Song: Conceptualization, writing—original draft and revising. Shichen Den: Writing—review and editing. Wei Zhang: Writing—review and editing. Mengjia Han: Writing—review and editing. Tianhang Lan: Writing—review and editing. Jingyun Ning: Collecting data and checking. Xiaokang Du: Collecting data and checking. XinHui Chen: Collecting data and revising. Haoming Lin: Writing—review and editing, supervision. Rui Zhang: Writing—review and editing, supervision. All authors have read and approved the final manuscript.

## Ethics Statement

The authors have nothing to report.

## Conflicts of Interest

The authors declare no conflicts of interest.

## Data Availability

The authors have nothing to report.
